# Continuing Education Workshops in Bioinformatics Positively Impact Research and Careers

**DOI:** 10.1371/journal.pcbi.1004916

**Published:** 2016-06-09

**Authors:** Michelle D. Brazas, B. F. Francis Ouellette

**Affiliations:** 1 Ontario Institute for Cancer Research, Toronto, Ontario, Canada; 2 Department of Cell and Systems Biology, University of Toronto, Toronto, Ontario, Canada; Whitehead Institute, UNITED STATES

## Abstract

Bioinformatics.ca has been hosting continuing education programs in introductory and advanced bioinformatics topics in Canada since 1999 and has trained more than 2,000 participants to date. These workshops have been adapted over the years to keep pace with advances in both science and technology as well as the changing landscape in available learning modalities and the bioinformatics training needs of our audience. Post-workshop surveys have been a mandatory component of each workshop and are used to ensure appropriate adjustments are made to workshops to maximize learning. However, neither bioinformatics.ca nor others offering similar training programs have explored the long-term impact of bioinformatics continuing education training. Bioinformatics.ca recently initiated a look back on the impact its workshops have had on the career trajectories, research outcomes, publications, and collaborations of its participants. Using an anonymous online survey, bioinformatics.ca analyzed responses from those surveyed and discovered its workshops have had a positive impact on collaborations, research, publications, and career progression.

B. F. Francis Ouellette is an Education Editor for *PLOS Computational Biology*

## Introduction

Continuing education workshops in bioinformatics offer a mechanism through which a researcher can quickly gain relevant computational skills in a defined topic. Many such programs exist around the world either through online, self-directed formats or through face-to-face, didactic, and hands-on formats (many of which are catalogued at mygoblet.org [1]).

While the reasons for seeking a continuing education workshop and gaining new skills may vary according to the research needs and career objectives of the participant, the goal of the workshop is to teach and hopefully develop a specific bioinformatic skill that can be applied to a research project. From an adult learning perspective, all aspects of the structure, organization, and delivery of the workshop should support the fulfillment of this goal [2].

**Fig 1:**
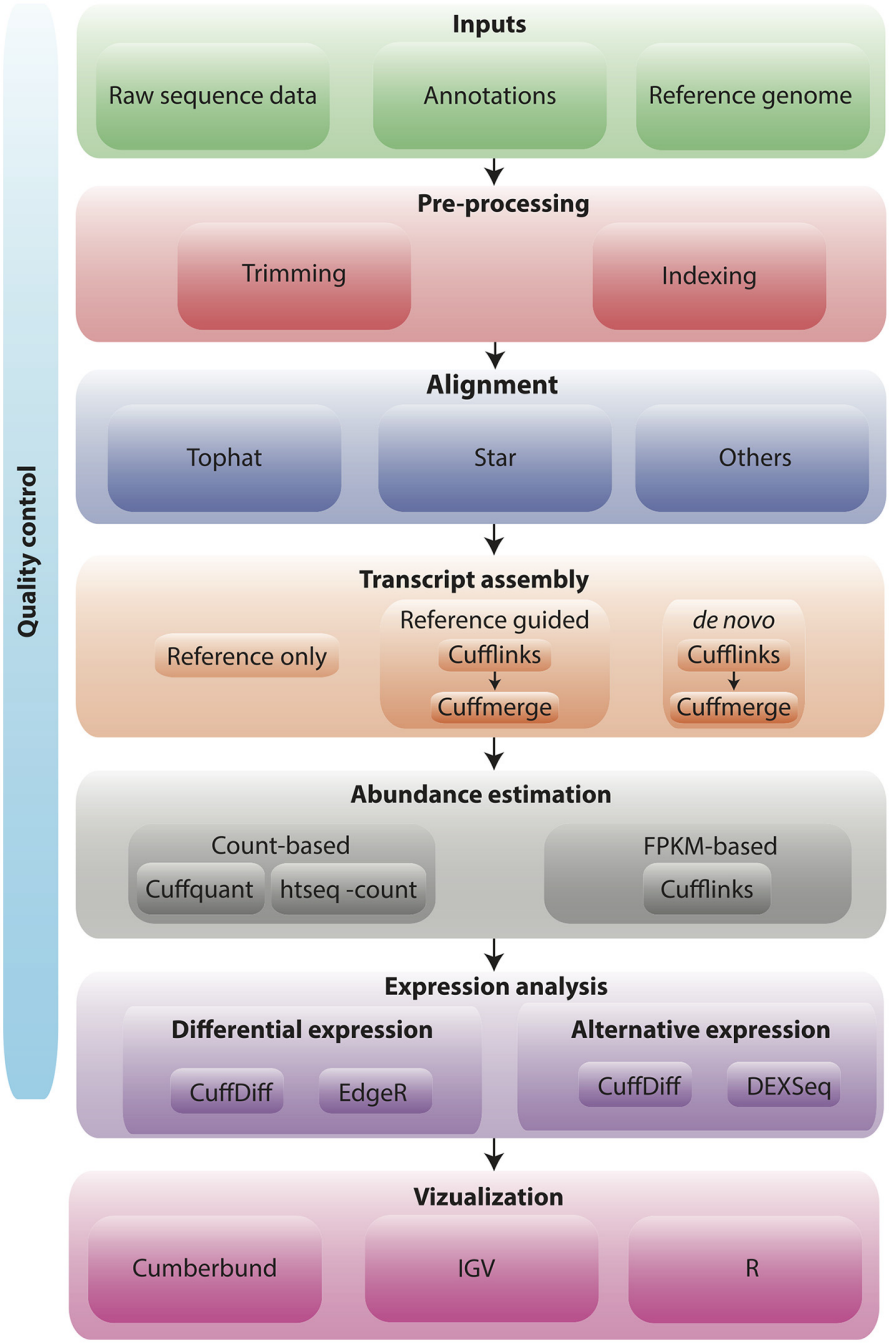
RNA-seq analysis workflow [3].

Since first offering continuing education workshops in 1999, bioinformatics.ca has worked to refine its workshop structure and delivery approach to ensure learning and skill development by its participants. Earlier bioinformatics.ca workshops (1999–2006) were broad, introductory topic courses in bioinformatics, genomics, or proteomics lasting between 1 week and 2 weeks per workshop with a maximum of 50 students each. Current workshops (2008–present) are short, intensive, advanced topic courses lasting between 2 days and 5 days per workshop with a maximum of 30 students each (see bioinformatics.ca for current topic offerings). All workshops, however, begin with either pre-workshop online exercises for skill review and/or selected articles for background preparation. Because participants are required to bring their own laptops, detailed tool installation instructions are also provided. Current bioinformatics.ca workshops follow a data analysis workflow model in which a particular data set, such as high-throughput sequence data from commonly used next-generation sequencing platforms, or analysis challenge, such as what to do with a gene list, is addressed by the workshop. An example workflow for the Informatics for RNA-seq Analysis workshop is provided in Fig 1 [3]. Within the workshop, faculty provide brief lectures on the data analysis concepts and bioinformatic approach as well as give an overview of the relevant bioinformatic tools. Faculty then work through use of the tools on a provided data set with the participants in a hands-on fashion. Participants are also given the opportunity to repeat the analysis workflow and tool use on a secondary data set during an integrated assignment or on their own data set where possible. Following the workshop, slide decks and videos are posted under a Creative Commons license (CC-BY-SA) on bioinformatics.ca (http://bioinformatics.ca/past-workshops) for the reference of participants and free access of everyone else.

While these approaches may help facilitate in-class learning of bioinformatic skills and support post-workshop access to learning resources, skill development is not formally measured during the continuing education workshops offered by bioinformatics.ca. Moreover, skill retention and application beyond the workshop environment has not been assessed by either bioinformatics.ca or any other known training program. The impact of our continuing education programs in bioinformatics is thus far unknown, yet it is an important indicator for development of the programs to achieve successful learning as well as for continued funding and resource allocation to these programs. With this impact gap in mind, bioinformatics.ca set out to gain some insight into the usefulness of its workshops over its history on research, publications, collaborations, and career progression.

## A First Measure of Impact

An online, anonymous form approach was taken for simplicity given that past workshop participants reside around the globe. The aim of the assessment was to gain insight into the impact of the workshop content and structure, organization and delivery on research outcomes, research collaborations, research publications, as well as career progression. Given that since 1999, bioinformatics.ca has taught over 2,000 participants across 60+ workshops (approximate figures for workshops held between 1999–2013), we also hoped to gather responses from a diverse cross section of workshops, career levels, and affiliations. Using the last known available email contact information for 1,276 of these participants (contact information was not known for the remaining participants), an invitation to participate in the online evaluation was sent to each participant via email at the end of February 2014 with a deadline of March 31, 2014, to complete the survey. Reminder emails were circulated 2 weeks prior to the close date and on the final day of the survey.

In addition to several general questions that sought information on institution, position, area of concentration, and workshop attendance, questions were centered around four major aspects where workshop impact was anticipated: (1) bioinformatics workshop skill retention and use in research; (2) collaborations; (3) research publications; and (4) skill/career progression (see S1 Survey for survey questions). Free-form optional textboxes were also used to allow respondents to provide answers not addressed by the available answer options. The impact evaluation form concluded with a few questions on whether continuing education workshops in bioinformatics were necessary in the future and in which areas of specialty were such workshops needed. These latter questions tie in with similar surveys previously conducted by the Society for Experimental Biology (SEB, http://mygoblet.org/about-us/goblet-events/sebgoblet-bioinformatics-workshop) and the Global Organisation for Bioinformatics Learning, Education and Training (GOBLET, personal communication).

## The Impact of Bioinformatics Training Is Positive

While it is unknown how many of the initial emails were not received due to inactive accounts or new email addresses, of the 1,276 potential participants, responses were received from 267 people, representing approximately a 21% response rate (only 256 responses [20% response rate] were used in the subsequent analyses as 11 surveys were incomplete). Of these survey respondents, 99 attended more than one workshop between 1999–2013. Responses received were from a variety of institutions, positions, and workshops (see S1–S4 Tables for demographics and S4 and S5 Figs for pairwise demographic analyses). All institutional categories (e.g., government, academia, industry, hospital, not-for-profit, etc.) were represented, with the majority of responses from those within academic institutions. A large variety of career positions from graduate students and post-doctoral fellows to principal investigators, professors, and directors was also represented, with a strong response rate seen from graduates and post-graduates across a breadth of disciplines in the academic setting (see S1–S3 Tables). The distribution of these affiliations and positions generally reflects those experienced during the 2008–2013 workshops. Despite not being able to contact approximately 40% of workshop participants and 79% of those contacted not being responsive, at least one response per workshop held since 1999 was received (with the exception of Patent Informatics). A table with the response rate per workshop is provided in S4 Table. Interestingly, the highest number of responses was seen for the most popular workshops. Overall, the response rate and breadth was beyond our expectation, given that we do not track participant movement beyond the workshop. Although the impact survey does not reflect a rigorous and comprehensive analysis of the impact of our individual training programs, it nevertheless provides some initial insight into the impact of bioinformatics.ca continuing education workshops. What is clear from this initial assessment is that the impact from even short course bioinformatics training is both real and positive.

**Fig 2:**
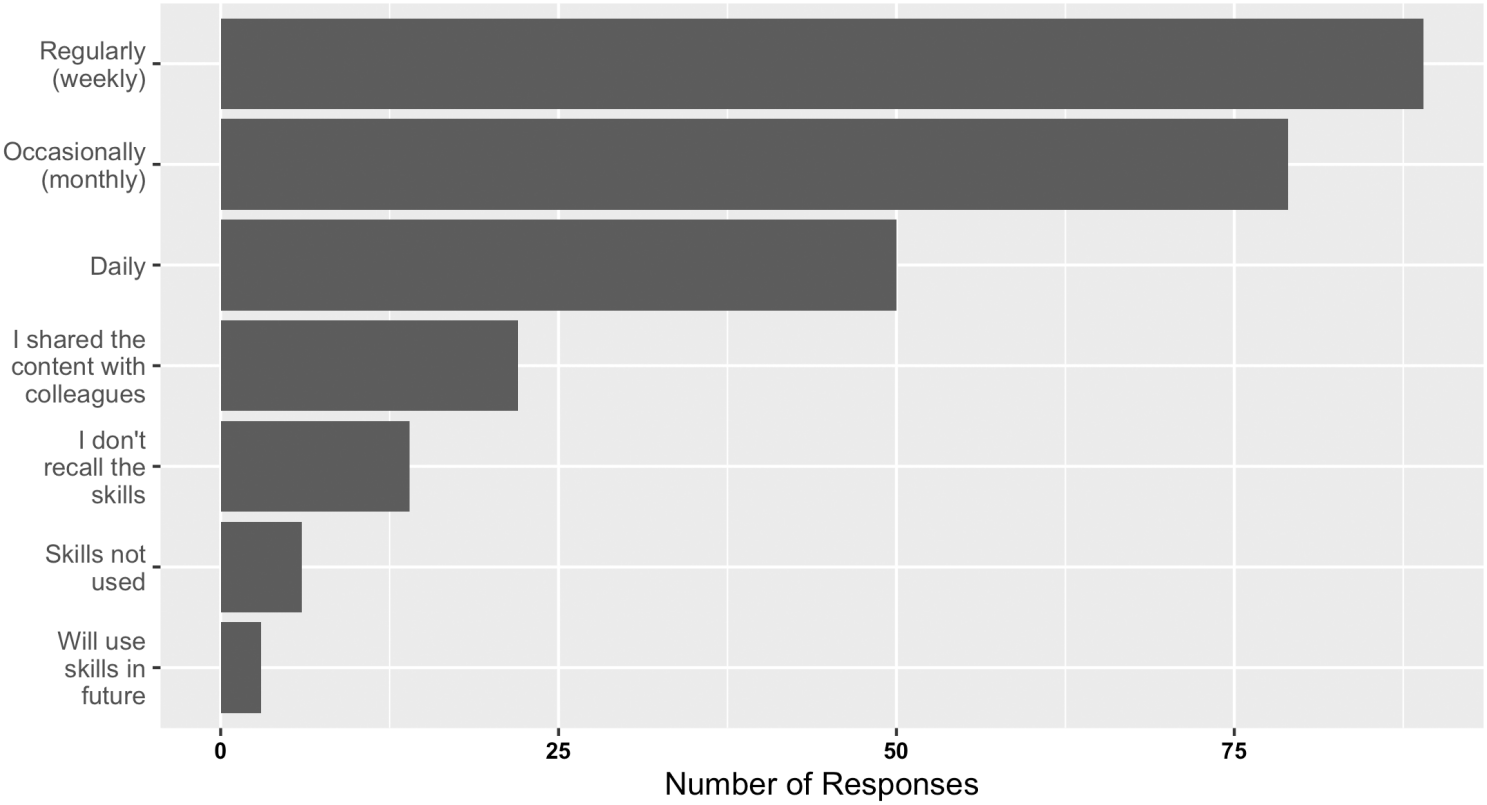
Responses received from past workshop participants as to their bioinformatics skill usage and retention post bioinformatics.ca workshop attendance.

Despite the significant time gap in gathering such feedback from workshop participants (since the first bioinformatics.ca offering in August 1999, in some instances), skills were generally retained and applied post-workshop with a fair amount of success (Fig 2 and S1–S3 Figs). Of the respondents, only 14 declared that they could not recall their bioinformatics skills. The majority used the bioinformatics skills gained through the workshops at least monthly, if not weekly or daily, an observation that was indistinguishable from the workshop attended (see S1 Fig showing workshop attended to subsequent skill usage). Importantly, this workshop skill usage was not limited to individuals identifying as bioinformaticians or to bioinformatics or computer science concentrations (see S2 and S3 Figs), demonstrating that workshop attendees across a broader spectrum of positions and research areas were able to acquire, retain, and use bioinformatics skills from our workshops. This positive frequency in workshop skill use implies bioinformatics skills taught during the workshop have had real impact. Because all bioinformatics.ca workshops incur a registration fee, it is possible that this positive skill retention and usage may be representative of a self-selected, highly motivated audience rather than a result of the workshop organization and delivery alone. Whether the registration fee was paid for personally or by a supervisor, grant, or company, a return on investment is expected. In the future, it may be worth polling the participant’s supervisor to learn if such return on investment was achieved from their perspective, as well as the participant’s.

Also important to note with respect to skill gain is that the reach of workshop impact extends beyond those attending the workshops, because many participants (22 respondents or 9%) also “shared (workshop content) with colleagues.” This content sharing, along with the open access publication of all bioinformatics.ca content (presentation slides and videos) on the website, makes measuring the true extent of training impact difficult.

**Fig 3:**
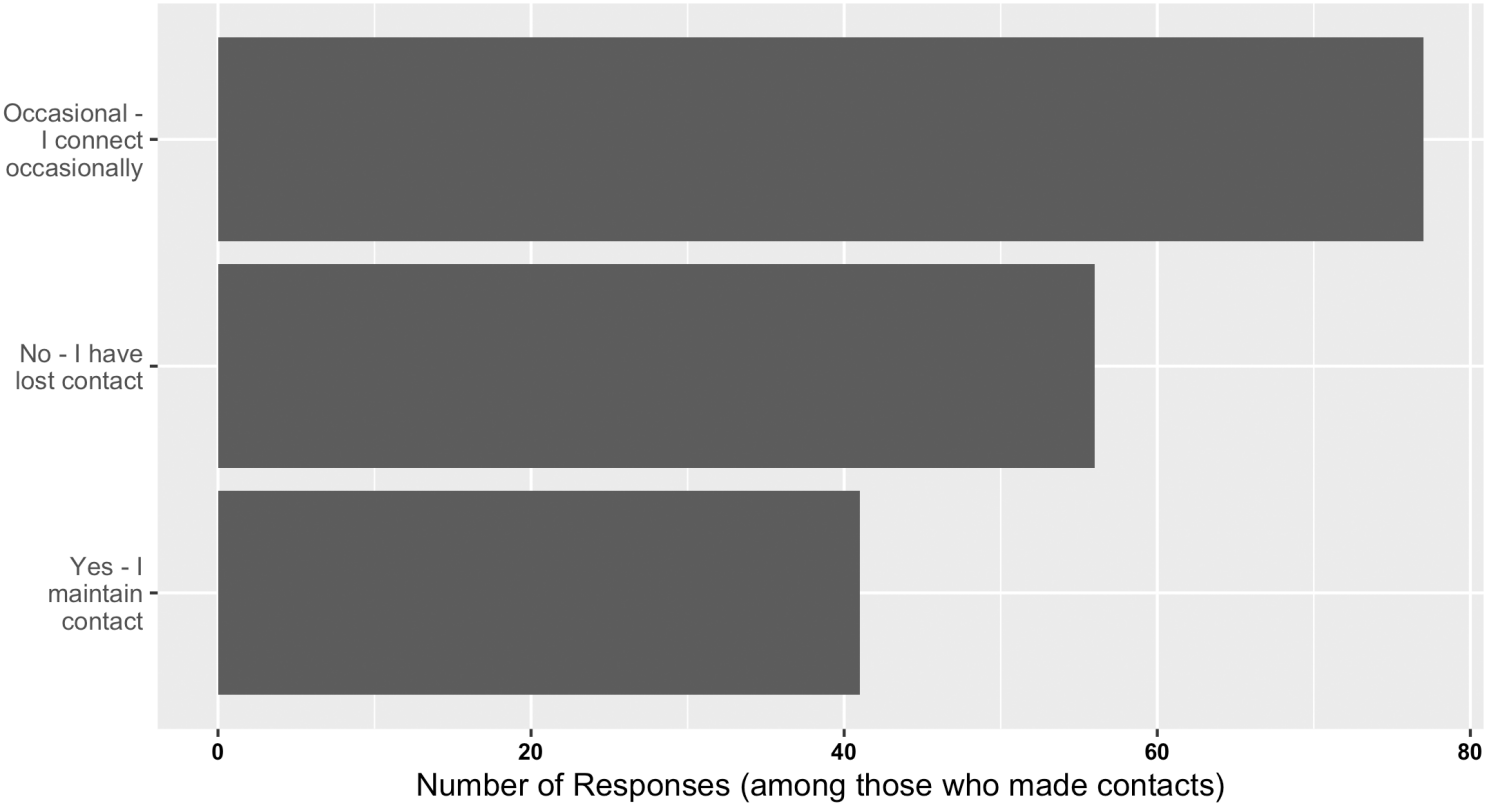
Responses received from past workshop participants on the frequency of correspondence with contacts from bioinformatics.ca workshops.

Further positive impact of bioinformatics.ca workshops was also evident on scientific networks, research, and publications. Of the respondents surveyed, 174 indicated that they met new contacts at the workshops. Not surprisingly, graduate students and post-doctoral/research fellows were particularly adept at making contacts (see S6 Fig). Of these, 105 respondents met one to three new contacts, 44 respondents met four to five new contacts, eight respondents met six to nine new contacts, and 17 respondents met over ten new contacts. Given an average workshop size of 25 (for workshops between 2008–2013; records incomplete for workshops from 1999–2006), this is a striking number of new contacts. Longer workshops, such as those offered between 1999–2006, may contribute to this observation because participants spent a greater length of time learning together. However, this is difficult to determine because the survey data does not distinguish the specific workshop a respondent attended or the workshop at which a contact was established. Thus, it is difficult to determine whether workshop length contributes to number of contacts met. Meeting and corresponding with new contacts represent very different efforts, so it was welcome to learn that the large majority of individuals who made new contacts through the workshops maintain regular to occasional correspondence (Fig 3).

**Fig 4:**
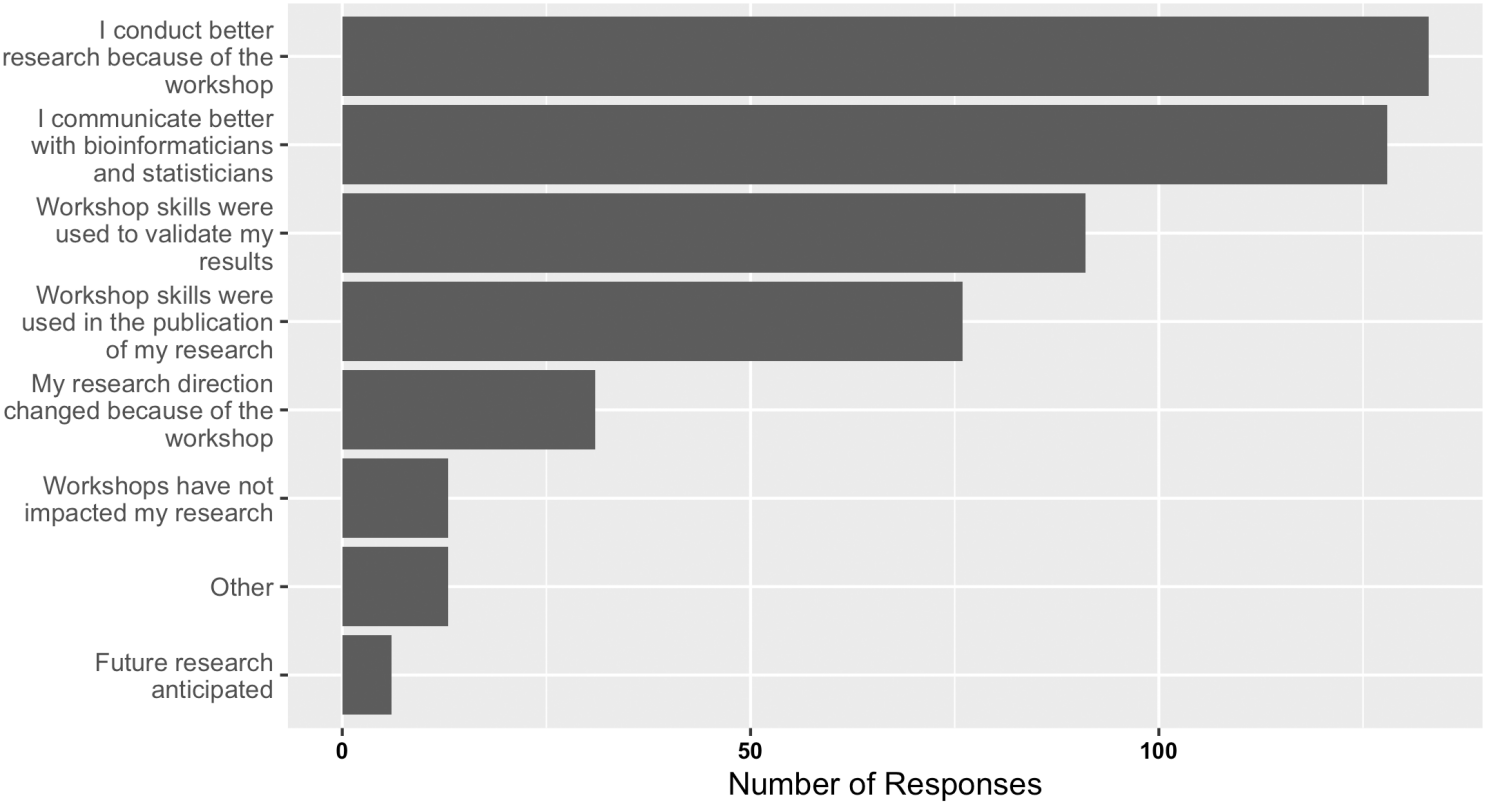
Responses received from past workshop participants on the impact of bioinformatics skills attained at bioinformatics.ca workshops on their research.

**Fig 5:**
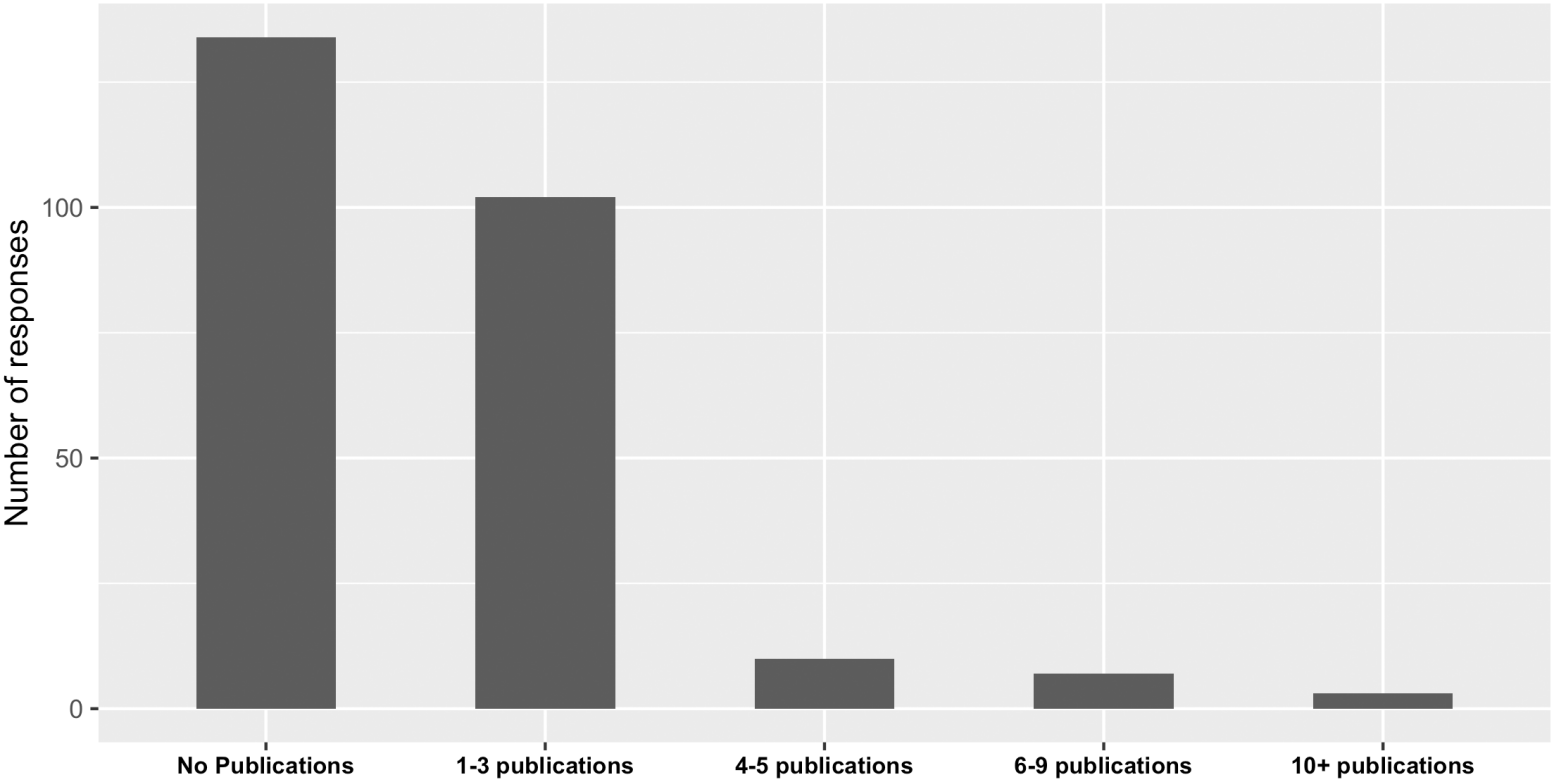
Responses received from past workshop participants on the publication impact of attending bioinformatics.ca workshops.

Most importantly, our impact assessment found that workshops had a positive impact on research (Fig 4 and S7 Fig) and publications (Fig 5 and S8 Fig). Because bioinformatic skills may impact numerous aspects of research, responses to how workshop skills affected research were not limited to a single answer. Responses ranged from helping communication with colleagues to helping validate results and publish research findings. Response categories that stand out with respect to positive impact include the following: (1) 128 respondents felt the workshops helped them communicate better with bioinformaticians and statisticians; (2) 133 respondents felt that they conducted better research because of participation in the workshops; (3) 91 respondents used workshop skills to validate results; and (4) 76 respondents used workshop skills for research publications. On closer inspection of these figures, while all career positions exhibited positive impact, graduate students and post-doctoral/research fellows experienced the most impact across the research measures (see S6–S8 Figs).

Further evaluation of those who used skills for publications revealed that workshops contributed to at least one publication for 122 respondents and four or more publications for 20 respondents across a diverse range of career positions (Fig 5 and S8 Fig). It will be interesting to explore the research areas and authors involved in the 65 PubMed identifiers (PMIDs) submitted in the optional text box associated with this survey question. Overall, the impact of bioinformatics.ca continuing education workshops on research was overwhelmingly positive.

**Fig 6:**
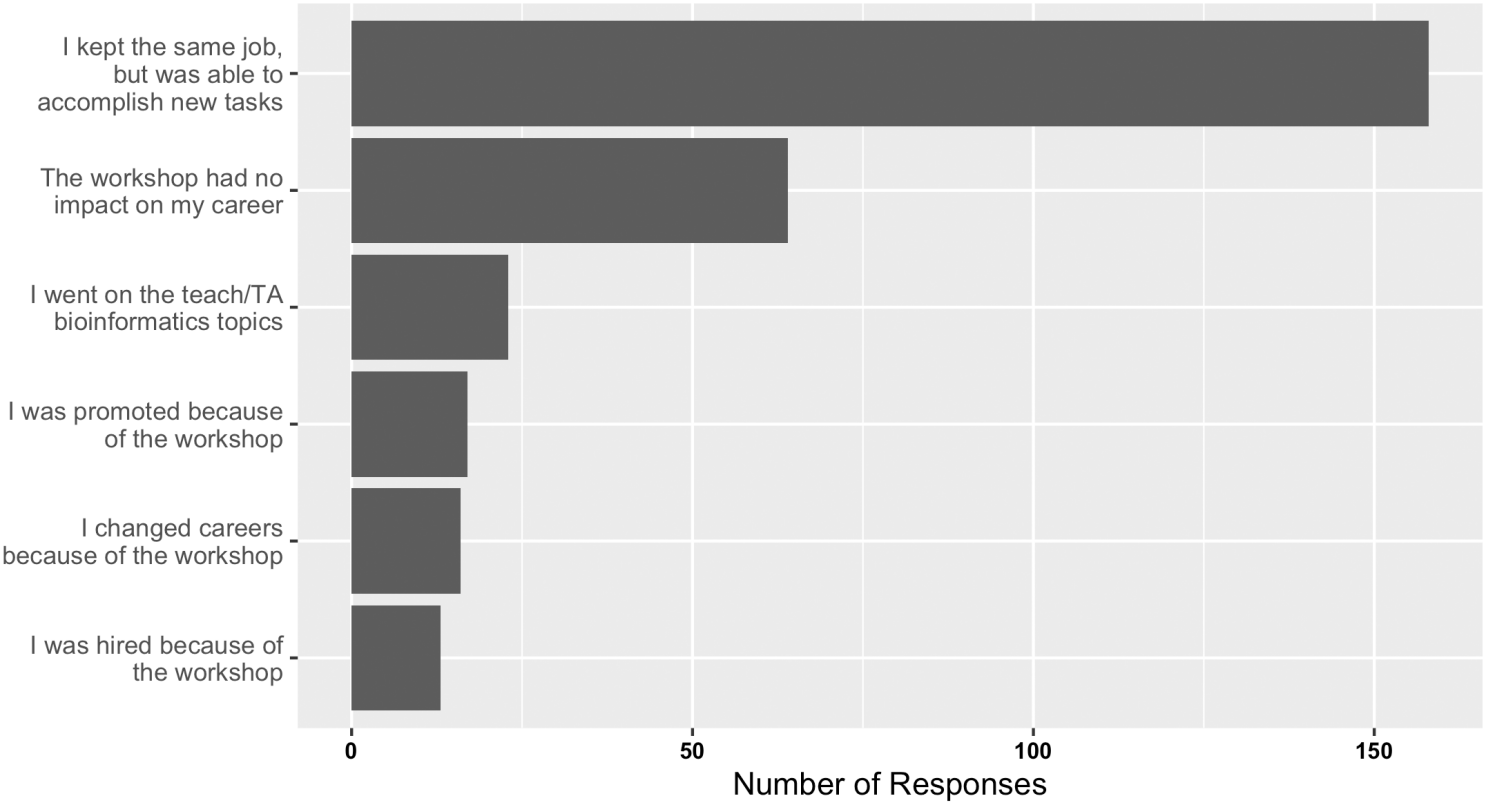
Responses received from past workshop participants on the career impact of attending Bioinformatics.ca workshops.

A positive impact of bioinformatics training was also noted for careers (Fig 6 and S9 Fig). Of the 256 respondents, 46 (18%) noted varying degrees of positive career impact, ranging from changing careers because of the workshops to being promoted or getting hired because of the skills gained through the workshops. Interestingly, observations for changing careers, being hired, or being promoted because of workshop skills were not limited to bioinformaticians whose skill sets are sought by employers, or by post-doctoral/research fellows whose hiring would be expected as their next career step (see S9 Fig). Instead, several career positions expressed such an impact. This kind of workshop impact is impressive, and several of the optional text responses in this category will be posted to bioinformatics.ca as testimonials of the value of the workshops on the participant’s career.

The general trends in this assessment point to a positive impact of bioinformatics.ca workshops on networks, research, publications, and career progression. What could not be quantified or adequately translated here in this article was the overwhelming number of supportive comments appended to each question within the optional textboxes. While the scored responses to each question highlighted the general success of bioinformatics.ca workshops on the areas queried, the optional written comments were more valuable evidence of successful workshop impact. Samplings of these comments are provided in Box 1.

**Box 1. Sampling of Written Comments**

“They were crucial to my gaining/keeping employment.”

“I believe that the workshops contributed to getting hired for my current position, which is largely bioinformatics focused.”

“Workshop skills help me discuss data analysis options with colleagues.”

“Finished my MSc project using tools in the workshop, and got my degree and a job after.”

“I got more interested in bioinformatics and much of my sabbatical I am taking is to learn more about it.”

“Contacts from the workshop helped me to create research collaborations and mentor bioinformatics students to accomplish research and publications I would not have otherwise attempted. I also began to take a more holistic view to biological data analysis and informatics.”

“The workshop has enabled me to propose new avenues of research now that I understand some of these topics better. The workshops gave me the fundamental background…”

## How to Track and Improve the Impact of Bioinformatics Training Programs

This assessment represents the first attempt at evaluating the impact of continuing education workshops in bioinformatics on various aspects of research. Knowing that bioinformatics.ca continuing education workshops have a positive impact on research outcomes is powerful and useful information. Looking forward, the question is how do we continue to track and improve upon the impact such continuing education programs have on research? Some tips for measuring impact are provided in Box 2. Firstly, assessments of impact will need to be refined and repeated on an individual workshop basis in order to provide workshop-specific feedback on skill retention, use, and impact on publications, collaborations, research progression, and career progression. Time since attending a workshop plays a large role in these various measures of impact, such that it would be useful to measure skill retention, skill use, and collaborations separate from measures on publication, research progression, and career progression. Based on our experiences with this initial impact survey, we would recommend evaluating skill retention and use at 6 months to 1 year post workshop and publication, research progression, and career progression at 3–5 years post workshop. The survey itself should also track changes in position since the workshop, as career level may also play a role in impact measures such as number of contacts and number of publications.

**Box 2. Tips for Measuring Impact**

- Actively track contact information for workshop participants if possible.
- Follow up on individual workshops to gain better workshop-specific data (rather than collectively surveying as was done here).
- Include measures that allow for comparison of impact survey results with workshop survey results (e.g., What was your position and concentration at the time of the workshop? What is your current position and concentration?).
- Set time frame(s) after the workshop over which to evaluate impact. Multiple time frames may be used depending on the impact being evaluated (e.g., shorter time frame for skill retention and use impact and longer time frames for publication and career progression impact).
- Collect as many responses as possible from participants in the original workshop. Link survey completion to an appreciation token meaningful for their research or career so you both gain value from the survey.
- Make collection of impact survey feedback an expected part of continuing education programs in bioinformatics.

Such impact surveys are only as valuable as the number of responses received, so when tracking individual workshop impact, it will be necessary to capture responses from as many of the original workshop participants as possible. This may prove difficult as researchers frequently move around and change email addresses, particularly as their careers progress. Requesting a permanent email address is one option for tracking participants beyond the workshop, as is more effective use of social media groups for each workshop. Beyond being able to reach workshop participants after the workshop, achieving participant buy-in to complete such impact surveys will also be a challenge. Playing on our own workshop funding vulnerability and sense of community was the strategy used by bioinformatics.ca to achieve this paper’s moderate 20% response rate. Linking survey completion to various other “carrots” such as bioinformatics compute resource credits or wiki access may help reach better response numbers.

While this initial impact survey demonstrated positive impact, it would be prudent for bioinformatics training programs to continuously consider workshop organization and facilitation techniques for ensuring and improving their impact beyond the classroom. Such improvements will likely come from better application of adult learning principles that address “supporting transfer of learning,” as well as aspects of “designing” and “facilitating” training [4]. For example, bioinformatics.ca has recently re-introduced integrated assignments into its workshops, in which students are given the opportunity to repeat the workshop’s analysis workflow on a second curated data set. This mechanism allows students to apply and reinforce their skills within the workshop’s learning environment, helping foster better skill retention and use. As another example of how to improve impact, bioinformatics.ca recently introduced into all of its workshops an introduction to the larger global bioinformatics community and resource space. At the beginning of each workshop, participants are encouraged to setup a BioStar account [5] and/or search through BioStar and other bioinformatics forums such as SEQanswers [6] and various R forums (https://r-dir.com/community/forums.html) for their particular bioinformatics questions. This activity is aimed at providing students with support mechanisms and strategies for dealing with bioinformatics problems they encounter beyond the classroom when workshop faculty are no longer available, which is often the biggest risk to continued usage of one’s newly acquired bioinformatics skills. Other methods for supporting transfer of learning beyond the classroom will help to ensure bioinformatics skills have a positive influence on research and careers. Most elements of the workshop organization were deemed extremely important or important by all positions of respondents (see S10–S15 Figs) with slightly lower importance given to workshop videos and wiki access, indicating that current workshop organization strategies should remain in place.

In summary, as a first evaluation of the impact of bioinformatics continuing education workshops on research, our bioinformatics.ca impact assessment results are highly encouraging. They indicate a positive influence on various aspects of research and point to the need to continue with such training opportunities. They also highlight the need to refine workshop content, delivery, and organization to ensure maximal impact of bioinformatics training on all aspects of research, which is the ultimate goal of all of our bioinformatics continuing education programs. We advocate for the inclusion of post-workshop impact assessments in all bioinformatics continuing education programs if the intent of our programs is truly to deliver on these lofty goals.

## Ethics Statement

No Institutional Review Board review was required for this work.

## Supporting Information

S1 Survey Survey questions used to assess impact of bioinformatics.ca workshops. (PDF)

S1 Fig Counts for workshop-attributed skill usage per workshop. (TIF)

S2 Fig Distribution of usage of workshop bioinformatics skills across current positions of survey respondents. (TIF)

S3 Fig Distribution of survey respondents by research concentration across frequency of workshop skill usage. (TIF)

S4 Fig Distribution of current research concentrations across current positions of survey respondents. (TIF)

S5 Fig Distribution of institution types across current positions of survey respondents. (TIF)

S6 Fig Distribution of workshop-attributed contacts across current positions of survey respondents. (TIF)

S7 Fig Distribution of workshop-attributed research impact across current positions of survey respondents. (TIF)

S8 Fig Distribution of workshop-attributed publications across current positions of survey respondents. (TIF)

S9 Fig Distribution of survey respondents by research position across frequency of career impact. (TIF)

S10 Fig Importance of face-to-face interactions to learning of bioinformatics. (TIF)

S11 Fig Importance of open access to slides to learning of bioinformatics. (TIF)

S12 Fig Importance of open access to exercises to learning of bioinformatics. (TIF)

S13 Fig Importance of availability of workshop scripts to learning of bioinformatics. (TIF)

S14 Fig Importance of access to workshop videos to learning of bioinformatics. (TIF)

S15 Fig Importance of access to workshop wiki to learning of bioinformatics. (TIF)

S1 Table Number of survey respondents per current position. (TIF)

S2 Table Number of survey respondents per current research concentration. (TIF)

S3 Table Number of survey respondents per current institution. (TIF)

S4 Table Number of survey respondents per workshop. (TIF)
