# Supplementary material for: Continuing Education Workshops in Bioinformatics Positively Impact Research and Careers
Source: PLoS Comput Biol. 2016 Jun 9;12(6):e1004916. doi: 10.1371/journal.pcbi.1004916 (PMC4900641; doi:10.1371/journal.pcbi.1004916)
Supplement: S1 Survey — (PDF) [file pcbi.1004916.s001.pdf]

Dear Canadian Bioinformatics Workshop Alumni,

Our funding agencies have requested that Bioinformatics.ca measure the impact of our introductory and advanced bioinformatics workshops.
We would like to use this online, anonymous survey to begin to capture how you have used or plan to use your bioinformatics training experiences.
Please help us meet our funding agency obligations by taking a moment to complete the following brief survey (5 minutes, 15 multiple choice questions). Thank you kindly for your assistance!

---

* Indicates a mandatory question

---

1. What is the best classification of your current institution?
- Academic
- Industry
- Government
- Hospital
- Not-for-Profit
- Other

Other (please specify):

2. What is your current position?
- Graduate Student
- Post-doctoral/Research Fellow
- Director
- Principal Investigator/Group Leader
- Lecturer
- Research-track Faculty
- Professor (Full, Associate, Affiliate)
- Technical Staff
- Bioinformatician
- Manager
- Other

Other (please specify)

3. What is your current area of concentration?
- Biomedical Sciences
- Plant Sciences
- Molecular Biology
- Marine Biology

- Microbiology
- Virology
- Biochemistry
- Biophysics
- Genomics
- Bioinformatics
- Medicine
- Mathematics/Statistics
- Computer Science
- Other

Other (please specify)

4. In my work, I currently use bioinformatics tools, databases or skills:

- Daily
- Regularly (weekly)
- Occasionally (monthly)
- Never
- Other

Other (please specify)

5. Which Canadian Bioinformatics Workshops did you attend?
Please select all that apply

- Introduction to Bioinformatics
- Genomics
- Proteomics
- Developing the Tools
- Pathway and Network Analysis of -omics Data (also called Interpreting Gene Lists)
- Informatics on High-throughput Sequencing Data
- Exploratory Data Analysis using R (also called Essential Statistics for Biology)
- Microarray Data Analysis
- Informatics and Statistics for Metabolomics
- Systems and Network Biology
- Clinical Genomics and Biomarker Discovery
- Bioinformatics for Cancer Genomics
- Ensembl EBI Browser and Toolkit
- Patent Informatics: Sequence & Chemical Databases
- Informatics for RNA-sequence Analysis
- Flow Cytometry Data Analysis using R

6. The bioinformatics skills I learned in the workshop(s) are currently used:

- Daily
- Regularly (weekly)
- Occasionally (monthly)

- I don't recall the skills
- I shared the content with colleagues
- Other

Other (please specify)

7. Did you meet new contacts at the workshops you attended?

- Yes
- No

If answered yes to question #7, then proceeded to question #8.
Otherwise, skipped to question #10.

---

8. How many contacts did you meet at the workshops you attended?

- 1-3 contacts
- 4-5 contacts
- 6-9 contacts
- More than 10

9. Do you still maintain contact with these individuals?

- Yes - I maintain contact
- Occasionally - I connect occasionally
- No - I have lost contact
- Other

Other (please specify)

10. How did attending the workshop(s) affect your career path?

Please select all that apply

- I changed careers because of the workshop
- I was hired because of the workshop
- I was promoted because of the workshop
- I kept the same job but was able to accomplish new tasks because of the workshop
- I went on to teach/TA bioinformatics topics
- Nothing happened to my career because of the workshop
- Other

Other (please specify)

11. How did the workshop(s) affect your research?

Please select all that apply

- I communicate better with bioinformaticians and statisticians
- I conduct better research because of the workshop
- My research direction changed because of the workshop
- Workshop skills were used to validate my results

- Workshop skills were used in the publication of my research
- Other

Other (please specify)

12. Approximately how many publications did the workshop(s) contribute to?

- No publications
- 1-3 publications
- 4-5 publications
- 6-9 publications
- More than 10

Please provide relevant PubMed IDs:

13. How relevant do you feel bioinformatics skills are to research today?

- Extremely relevant
- Very relevant
- Relevant
- Not really relevant
- Not required

14. How important do you feel the following workshop components are to learning bioinformatics?

Possible ratings:

- Extremely relevant
- Very relevant
- Relevant
- Not really relevant
- Not required

Components to rate:

- Face to face TA/Faculty interaction
- Open access to workshop slides
- Open access to workshop exercises
- Availability of workshop scripts
- Access to workshop video recordings
- Access to the workshop wiki beyond the workshop

Comments:

15. Should Bioinformatics.ca continue to offer the Canadian Bioinformatics Workshops?

- Yes
- No

Comments:

16. Looking ahead, what workshops or content would you like to see?
