# Supplementary figures and images for: Continuing Education Workshops in Bioinformatics Positively Impact Research and Careers
Source: PLoS Comput Biol. 2016 Jun 9;12(6):e1004916. doi: 10.1371/journal.pcbi.1004916 (PMC4900641; doi:10.1371/journal.pcbi.1004916)

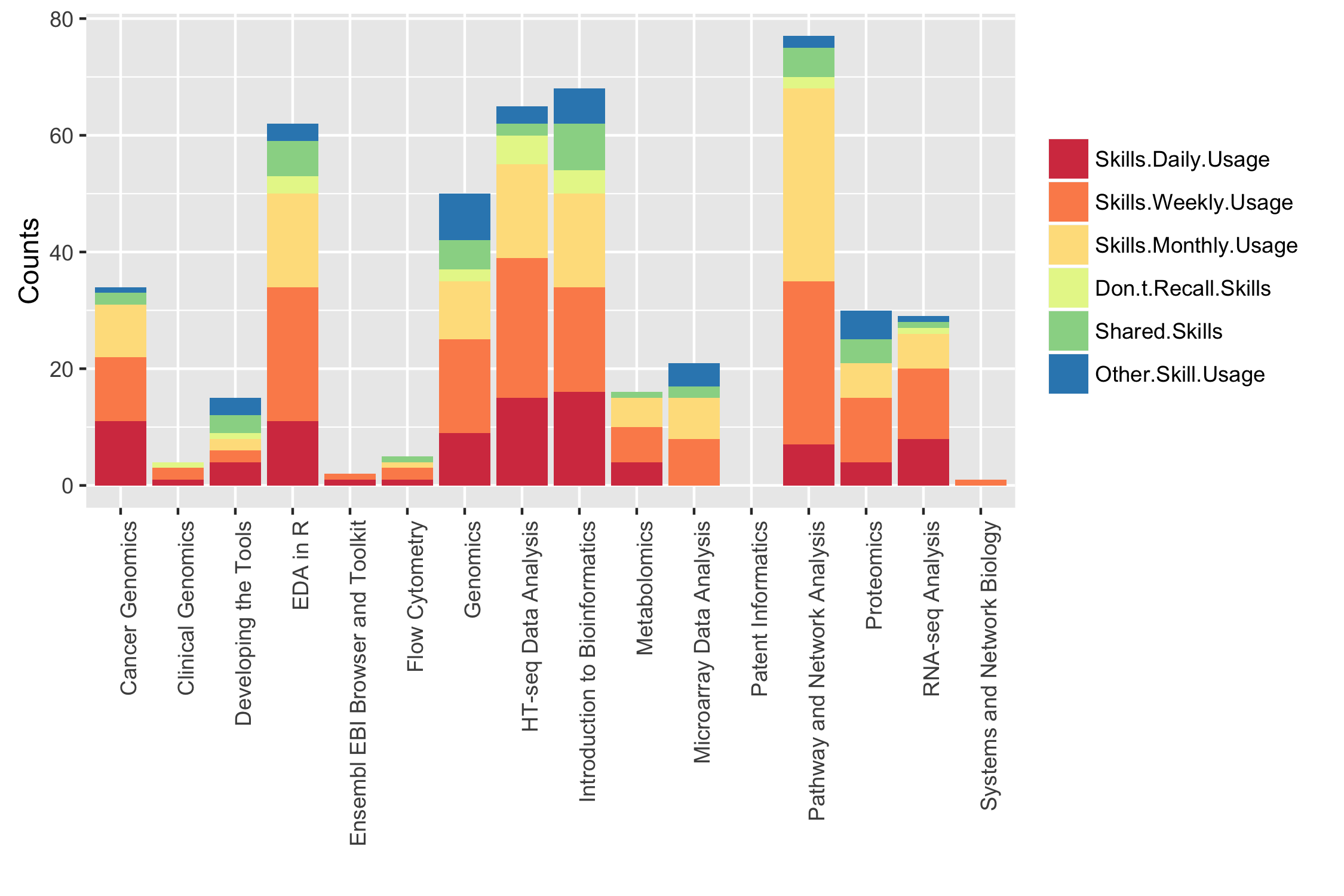

Supplement: S1 Fig — (TIF) [file pcbi.1004916.s002.tif]

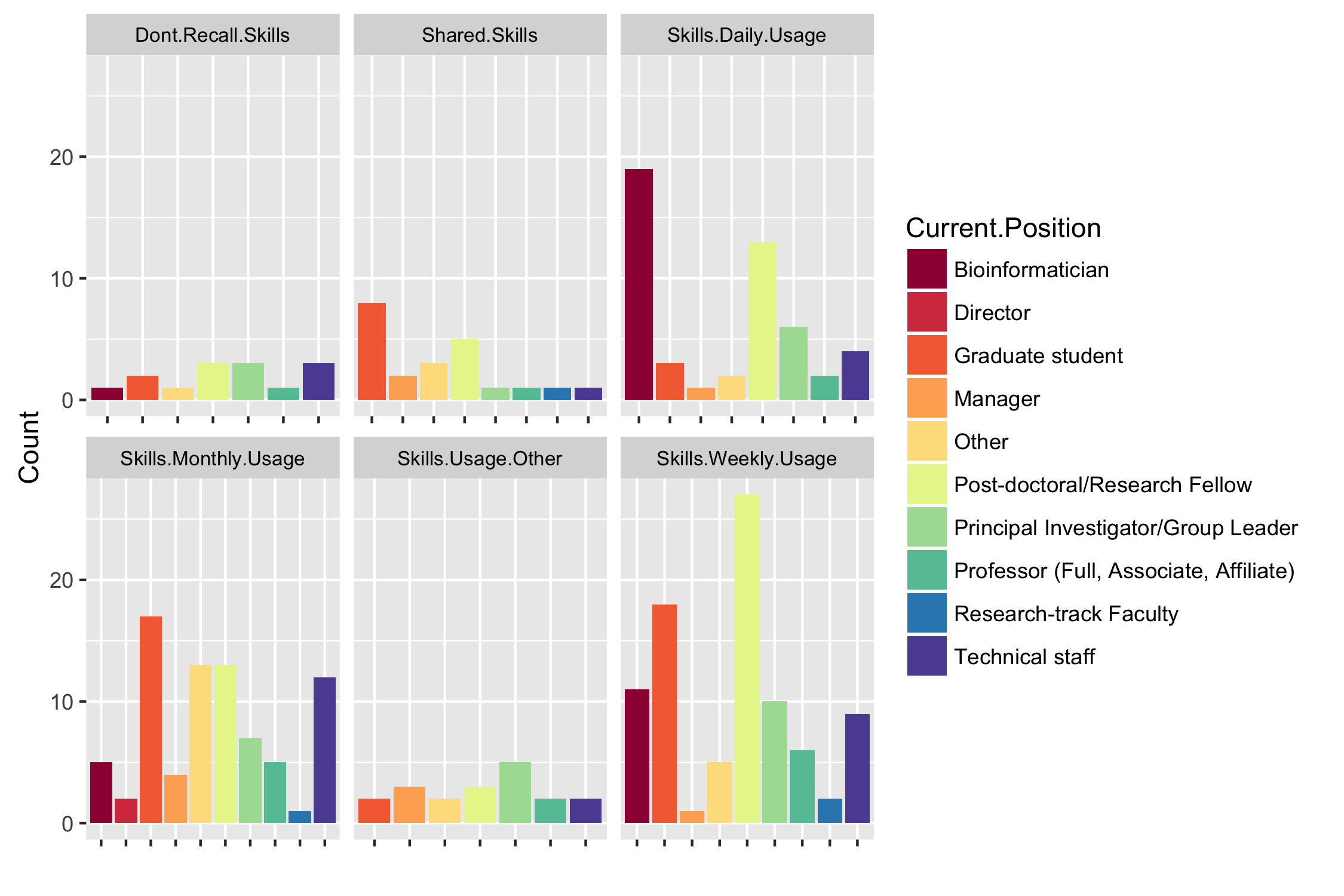

Supplement: S2 Fig — (TIF) [file pcbi.1004916.s003.tif]

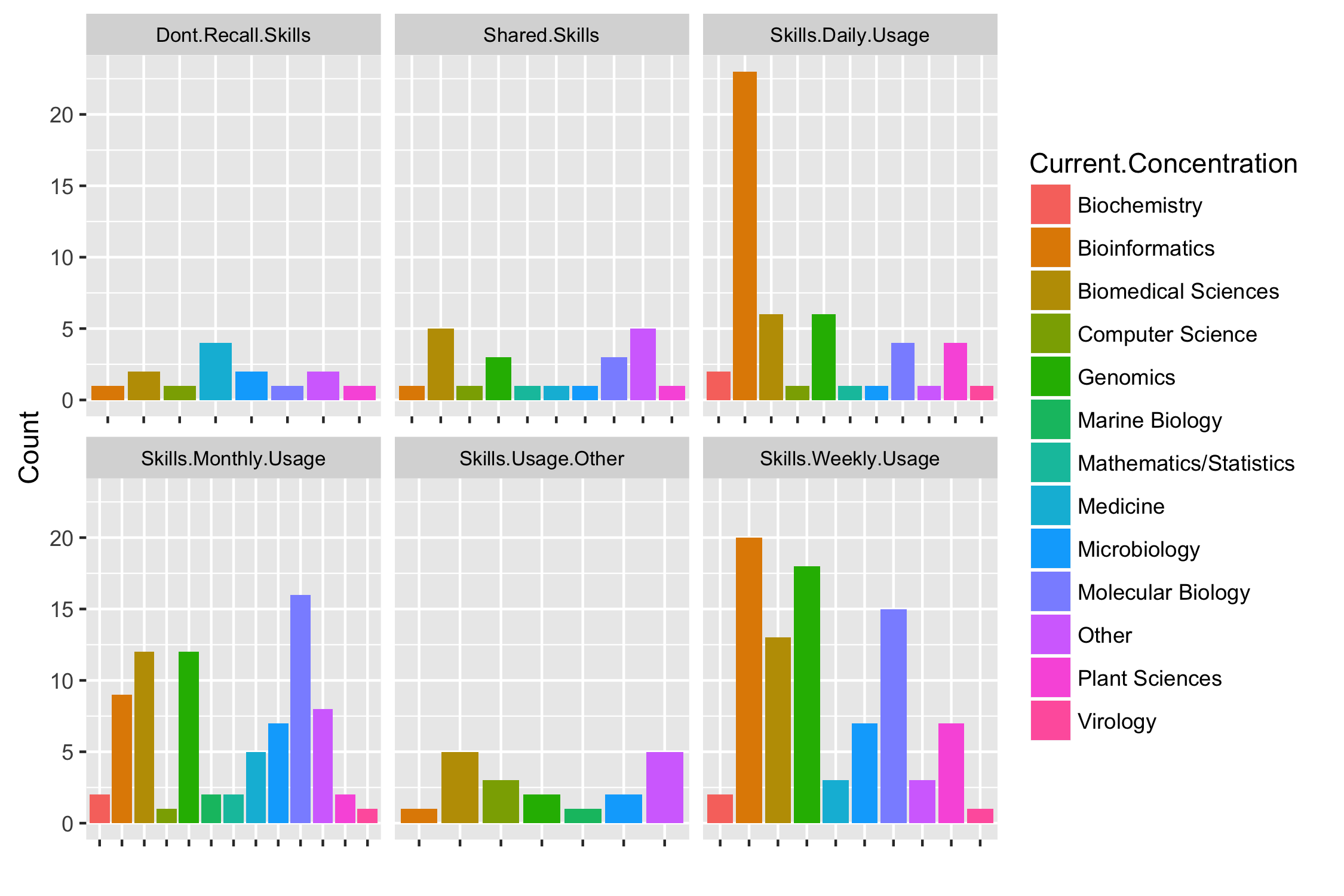

Supplement: S3 Fig — (TIF) [file pcbi.1004916.s004.tif]

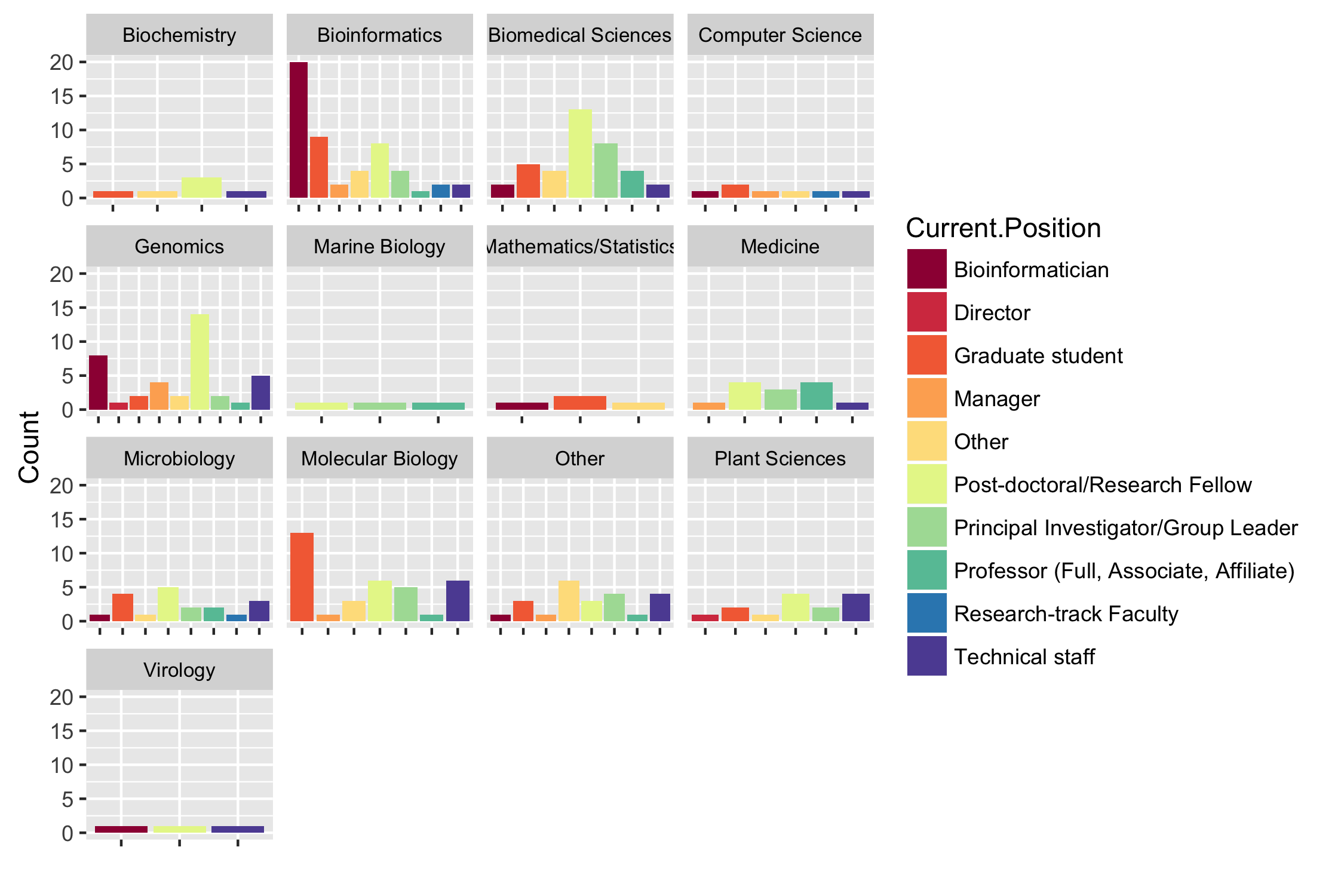

Supplement: S4 Fig — (TIF) [file pcbi.1004916.s005.tif]

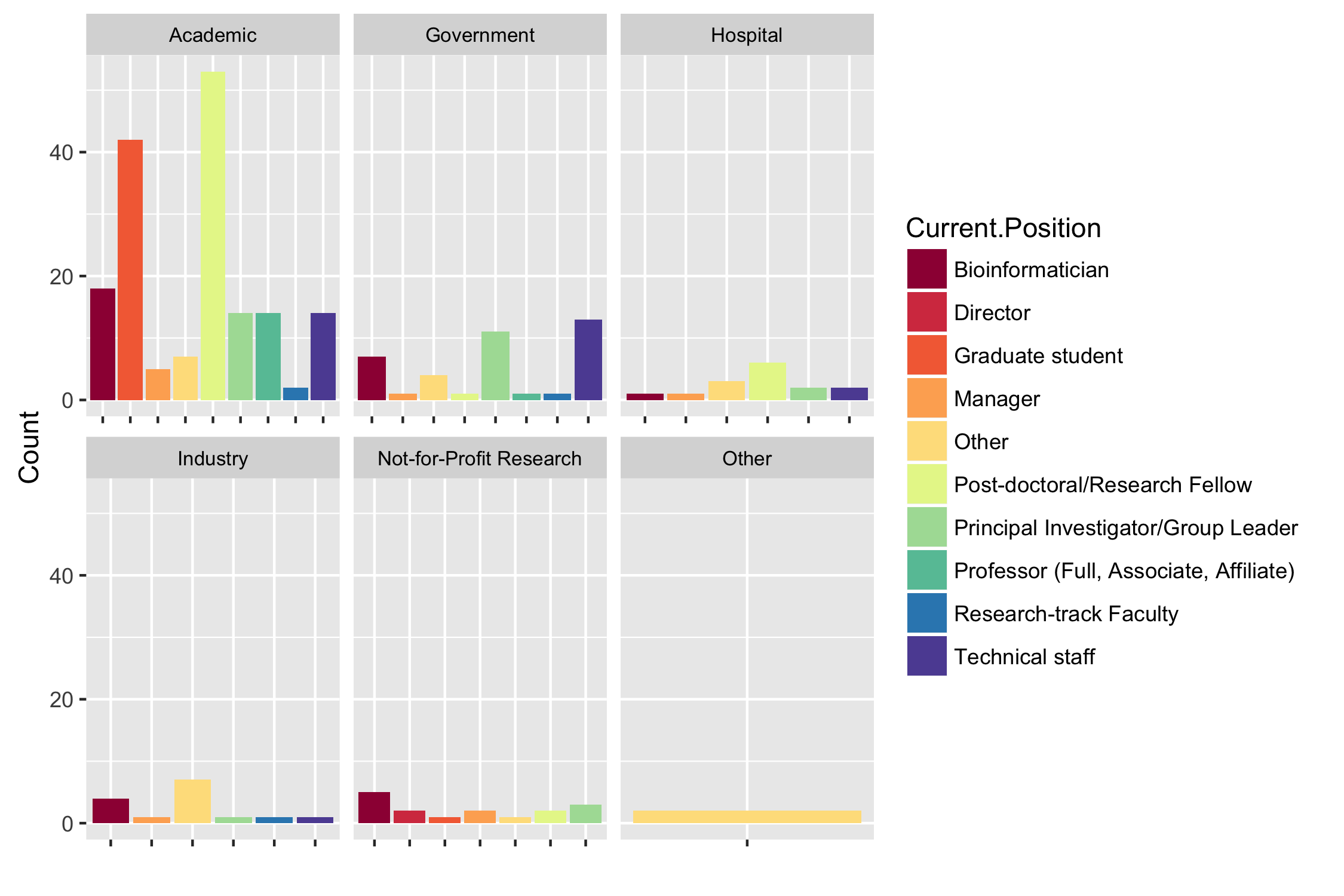

Supplement: S5 Fig — (TIF) [file pcbi.1004916.s006.tif]

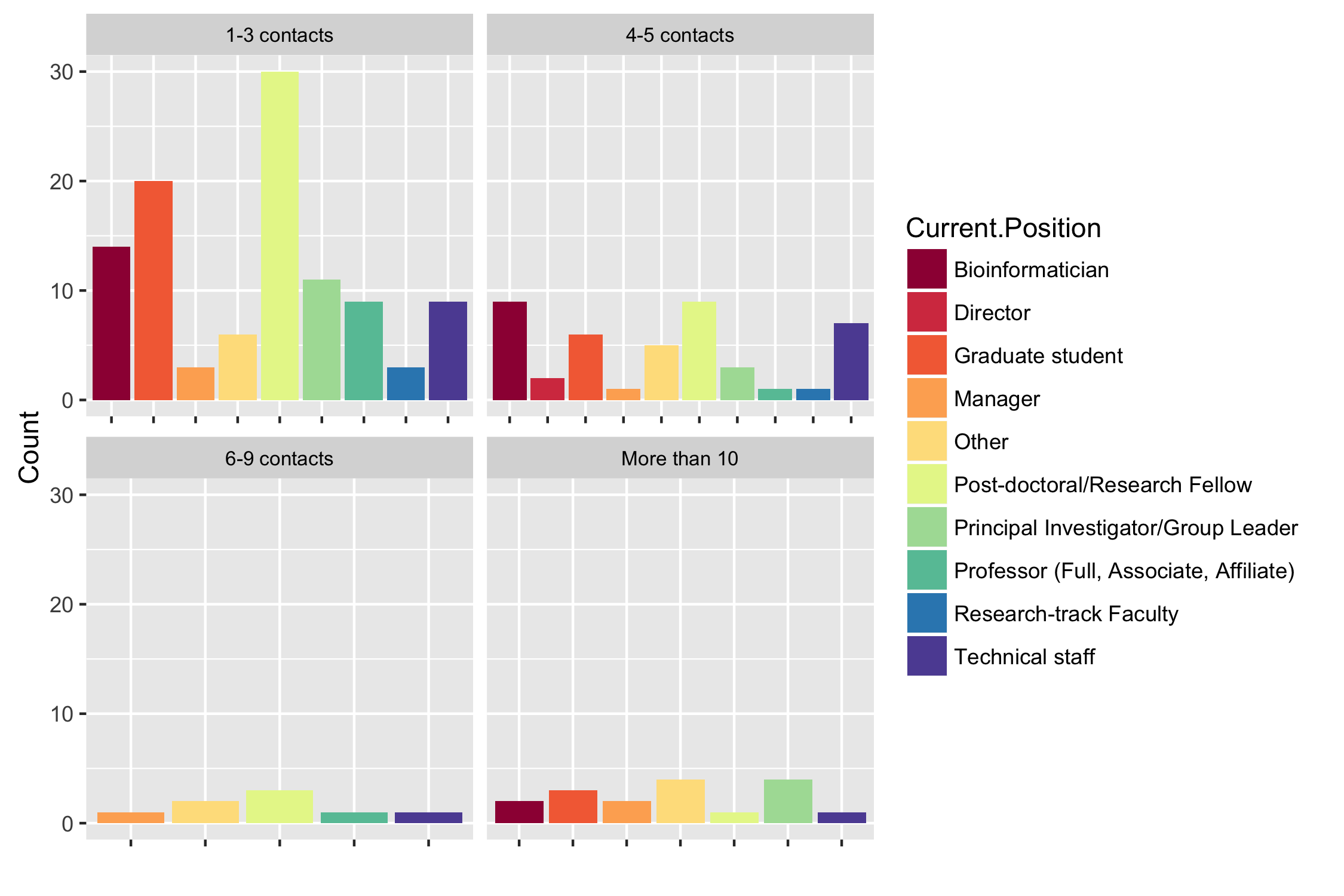

Supplement: S6 Fig — (TIF) [file pcbi.1004916.s007.tif]

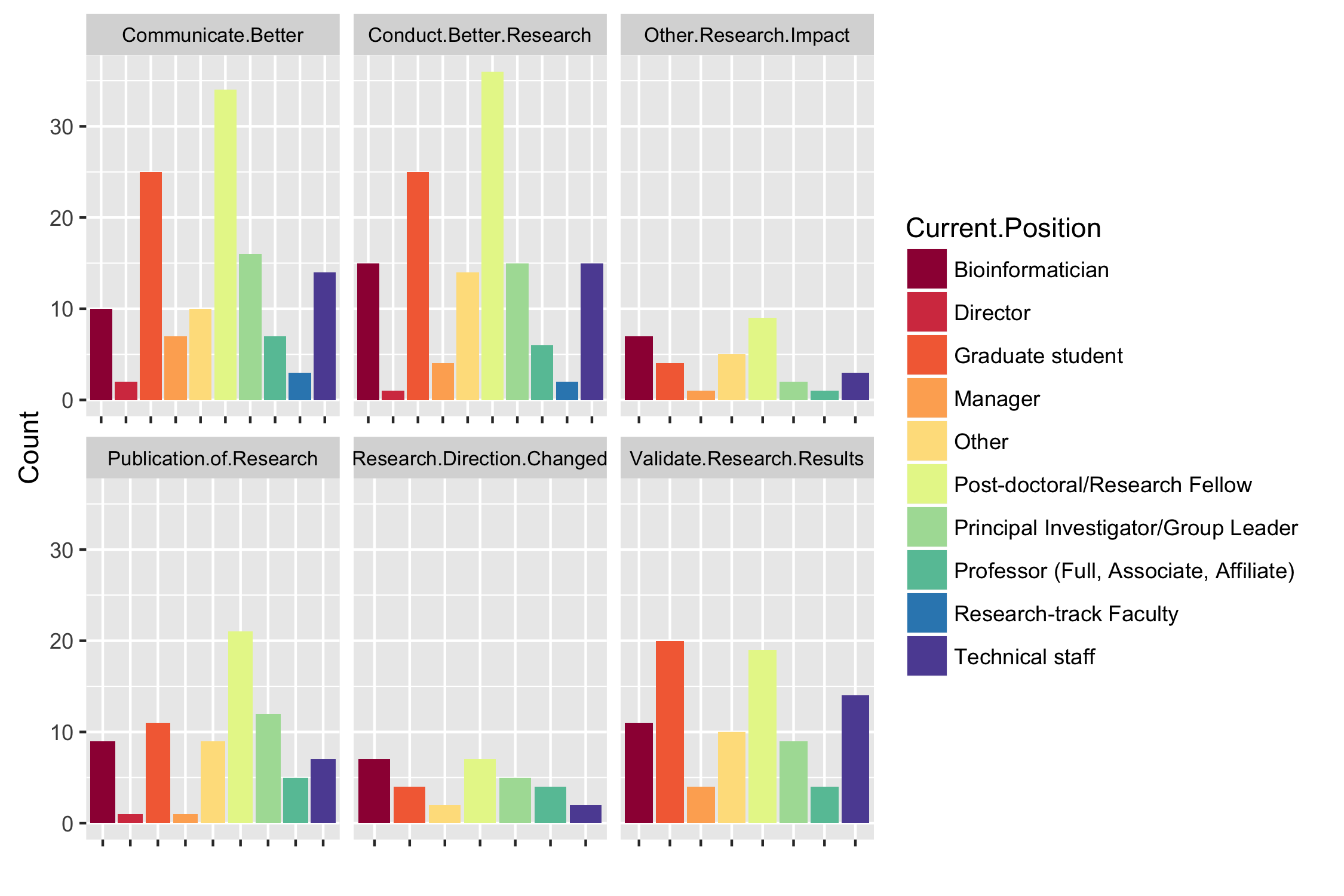

Supplement: S7 Fig — (TIF) [file pcbi.1004916.s008.tif]

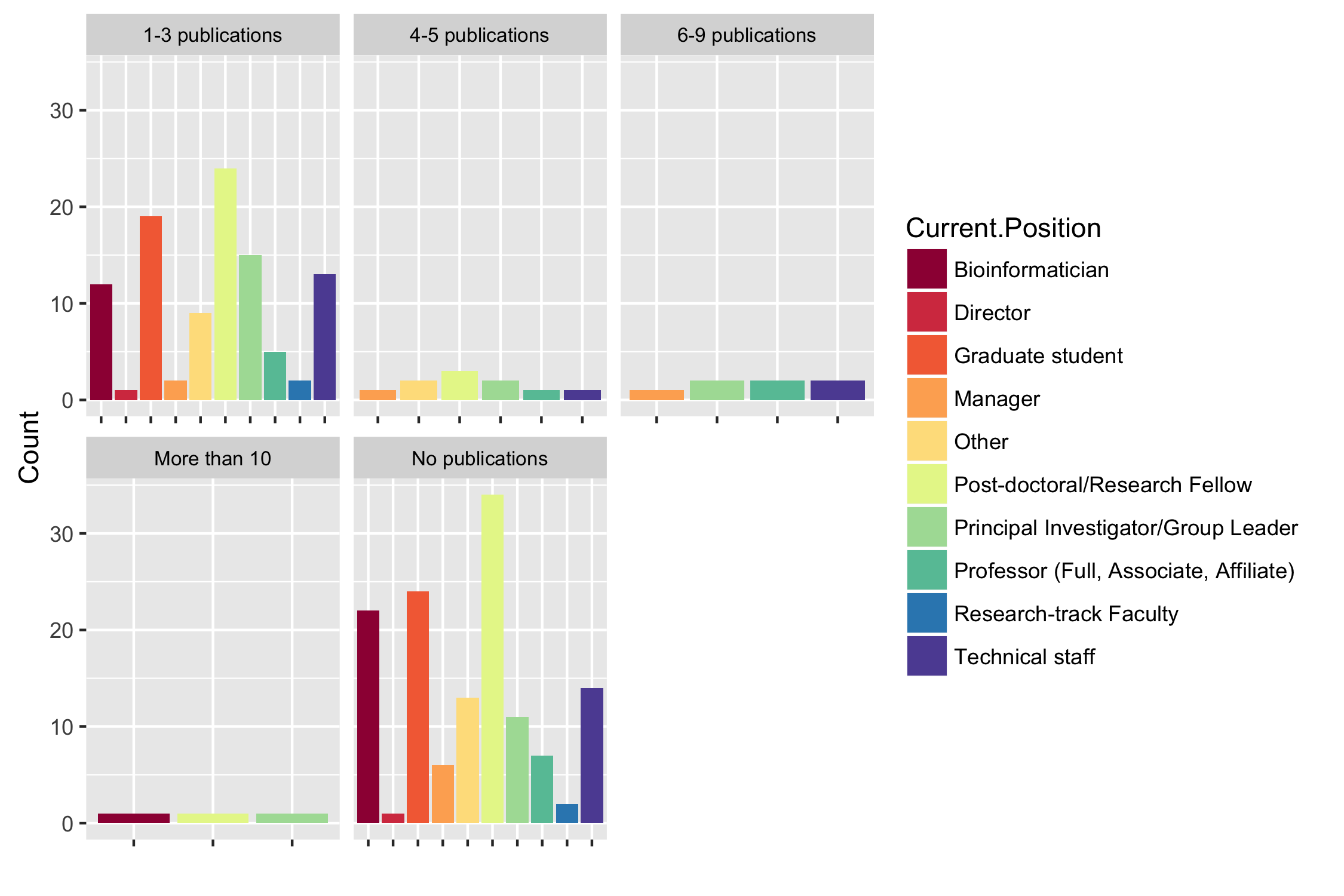

Supplement: S8 Fig — (TIF) [file pcbi.1004916.s009.tif]

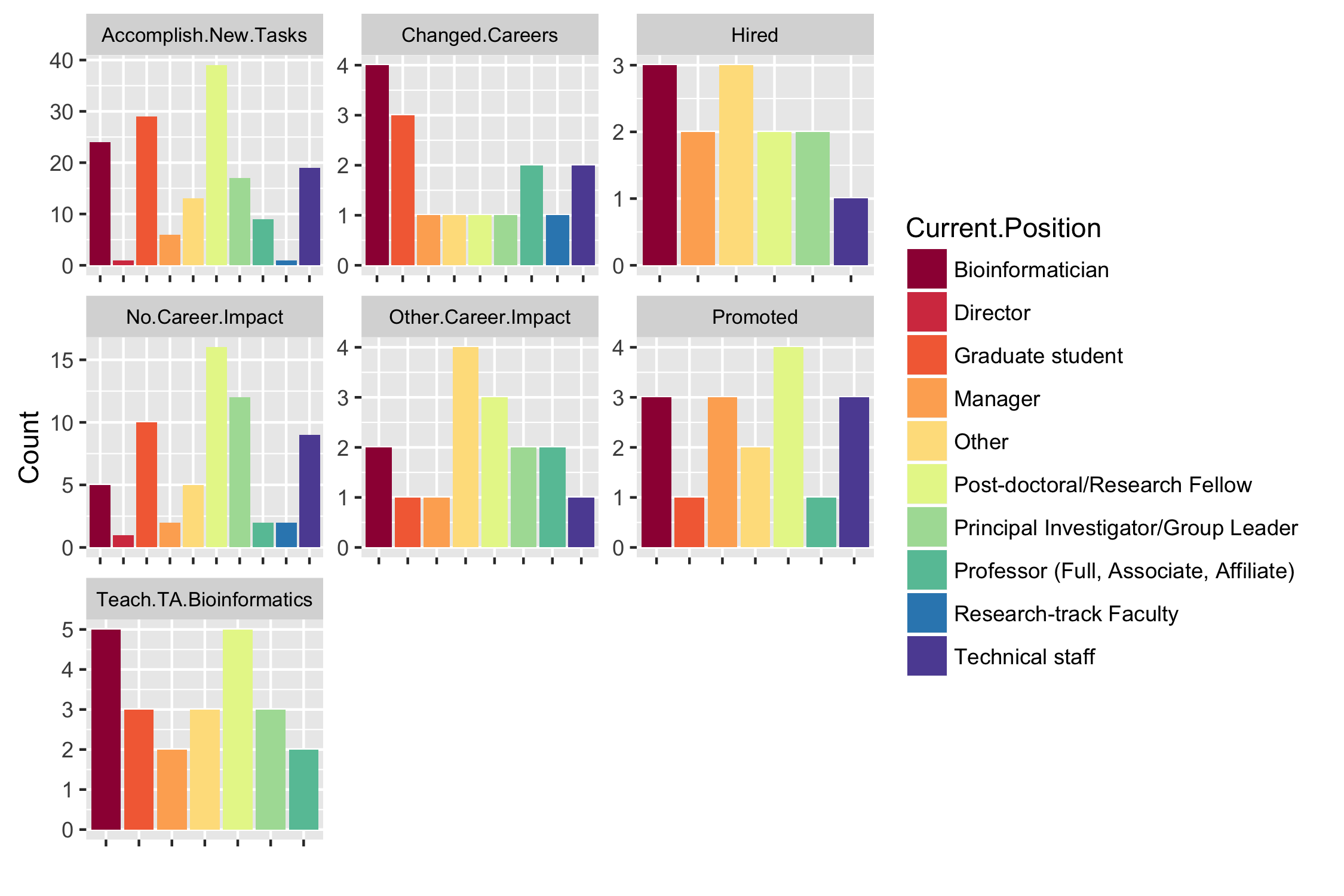

Supplement: S9 Fig — (TIF) [file pcbi.1004916.s010.tif]

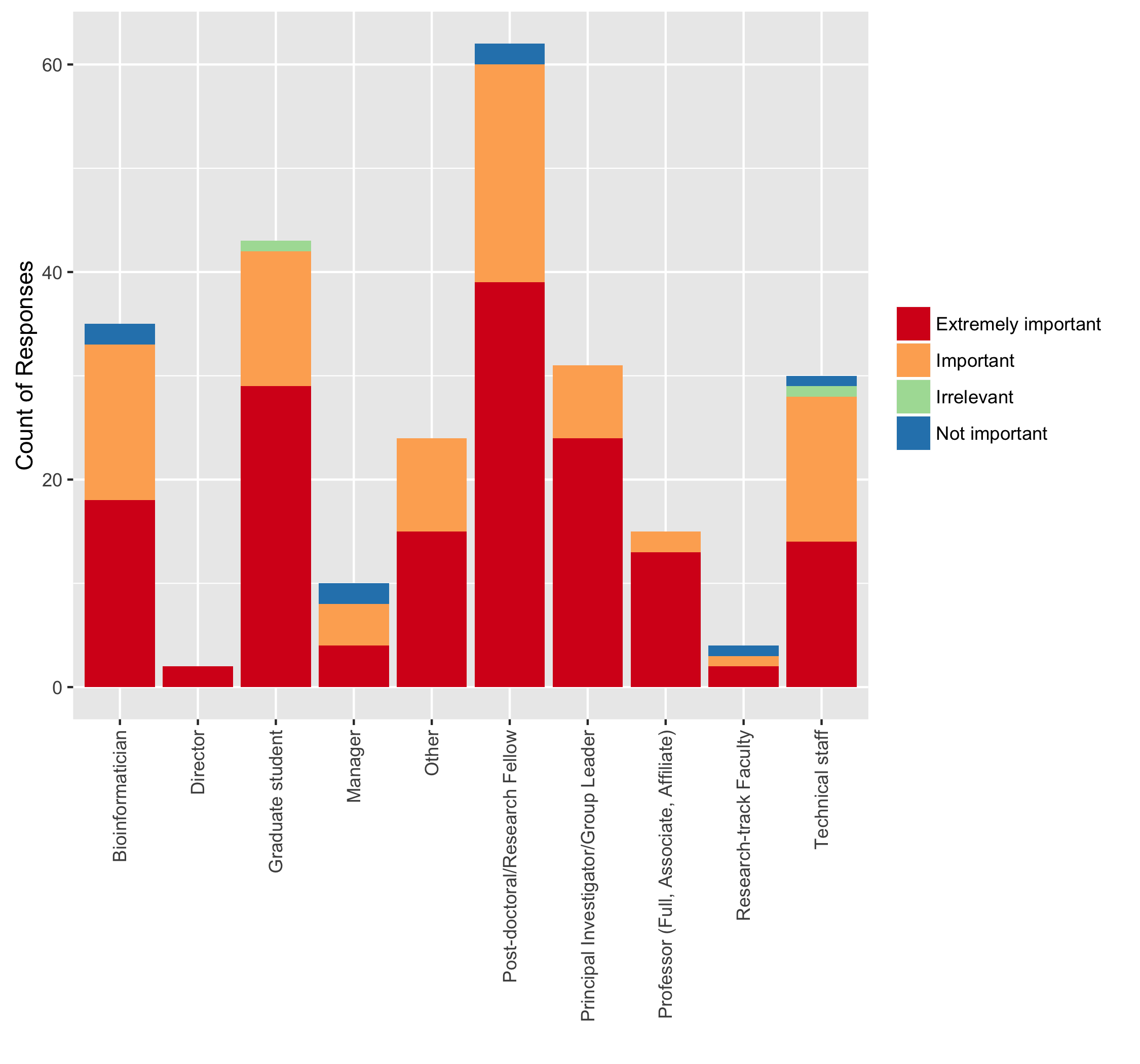

Supplement: S10 Fig — (TIF) [file pcbi.1004916.s011.tif]

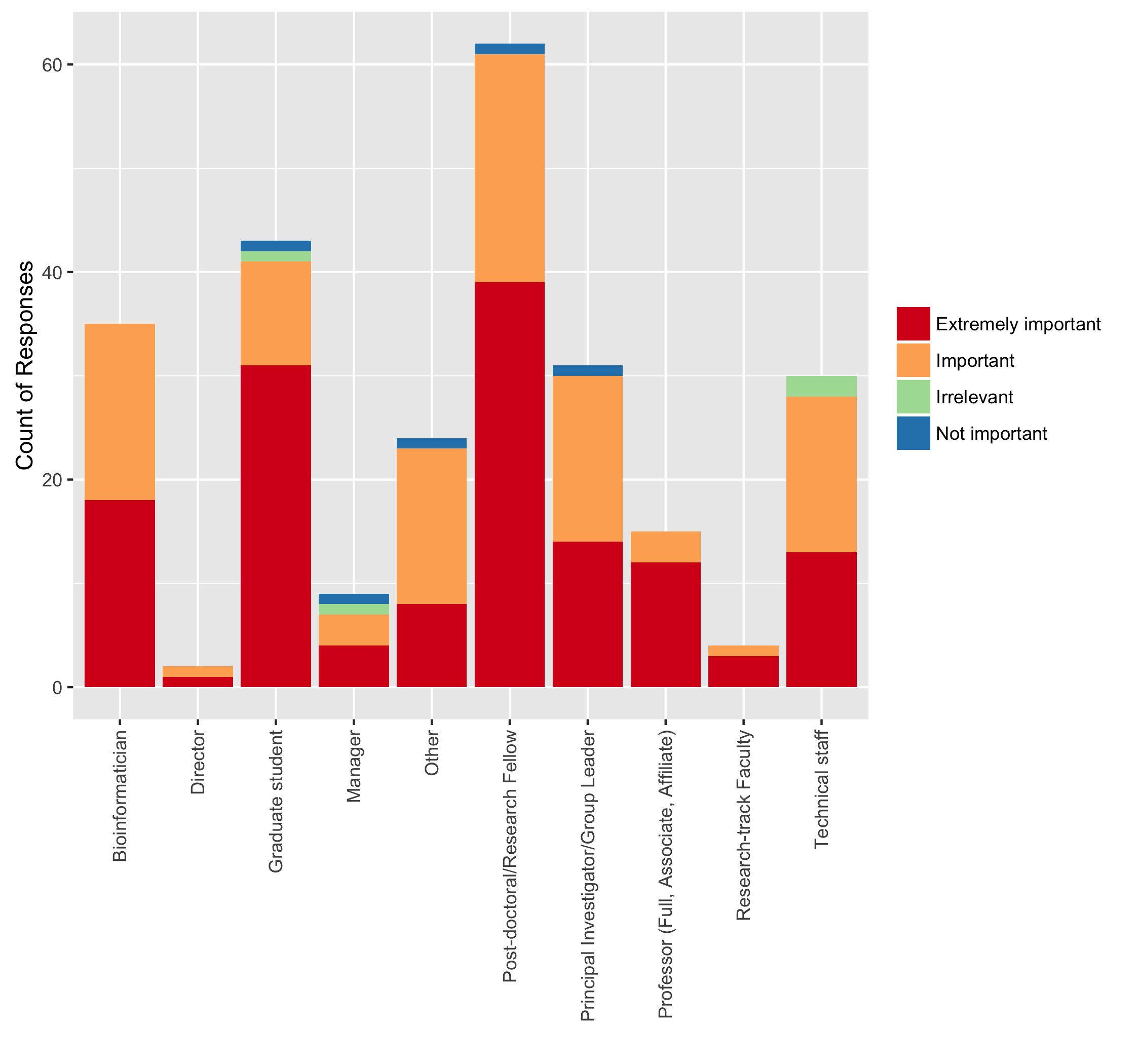

Supplement: S11 Fig — (TIF) [file pcbi.1004916.s012.tif]

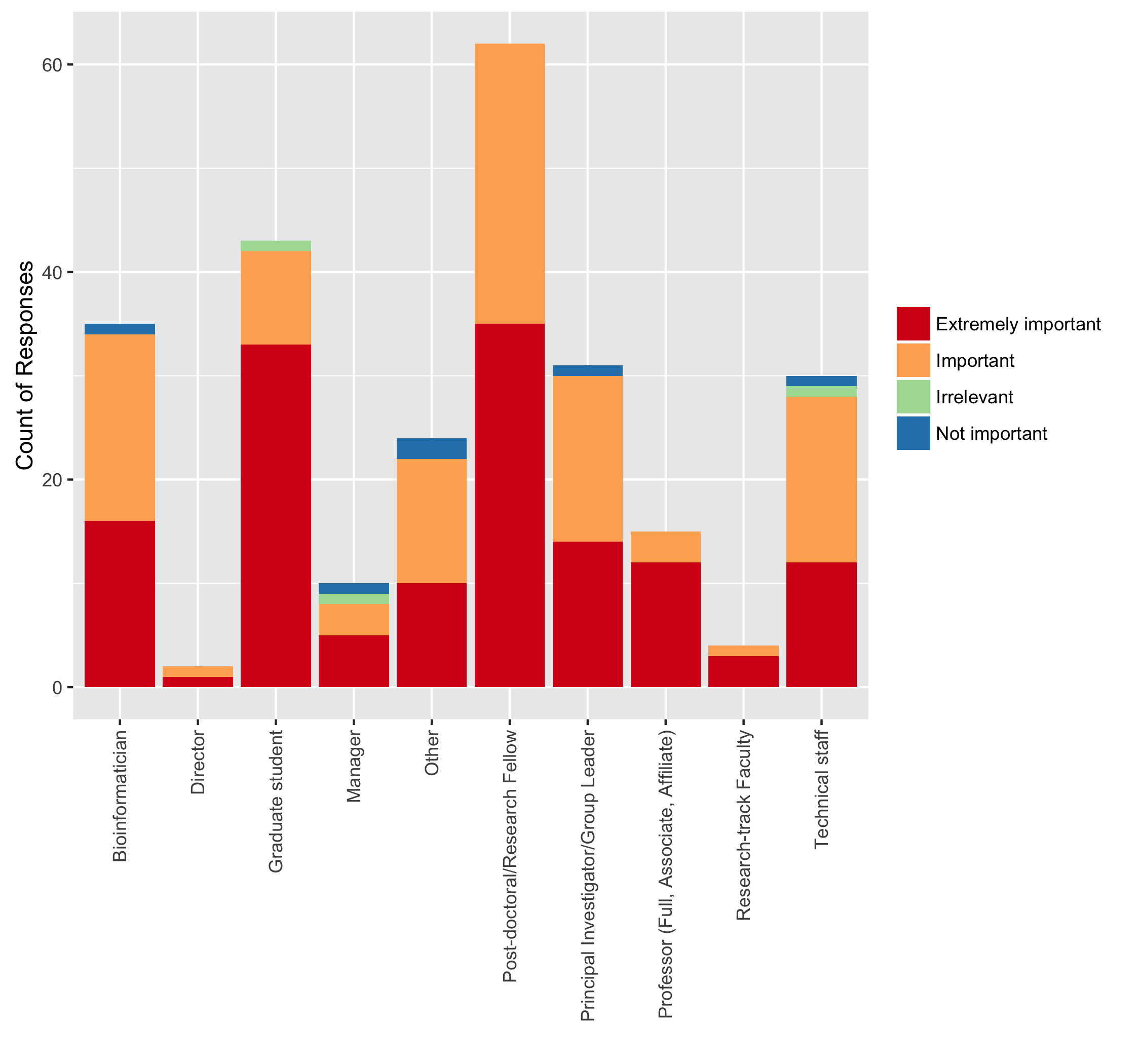

Supplement: S12 Fig — (TIF) [file pcbi.1004916.s013.tif]

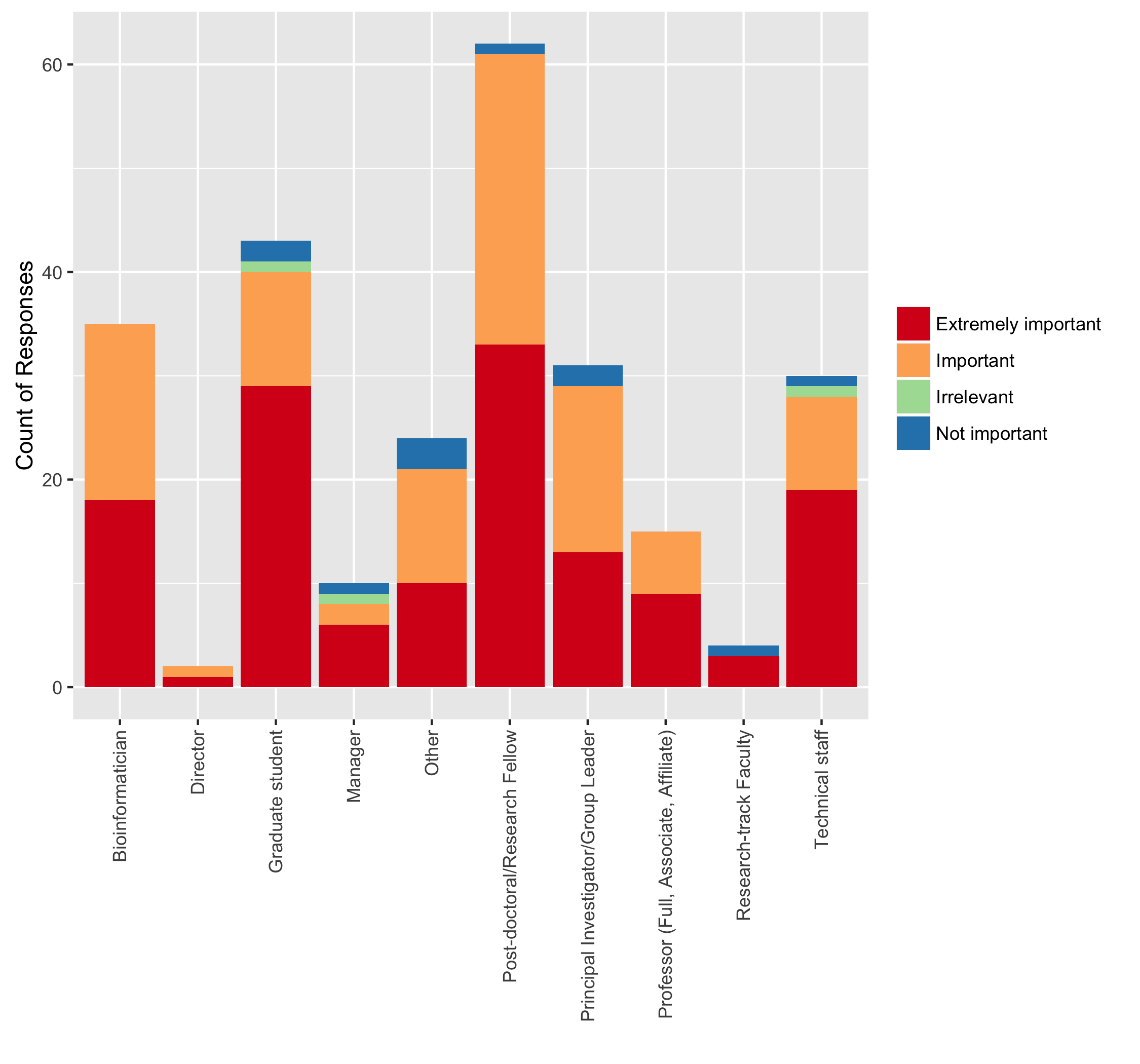

Supplement: S13 Fig — (TIF) [file pcbi.1004916.s014.tif]

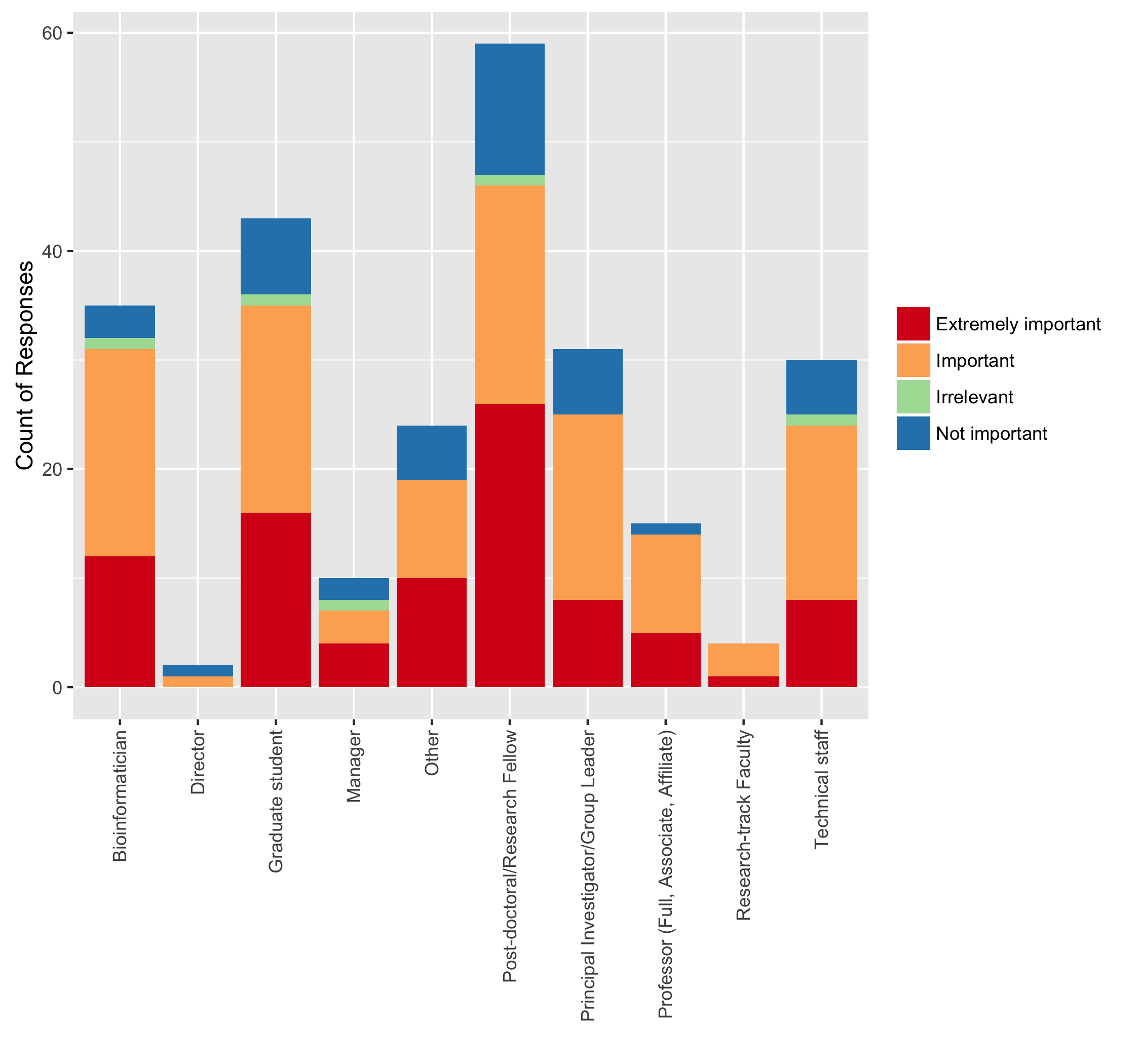

Supplement: S14 Fig — (TIF) [file pcbi.1004916.s015.tif]

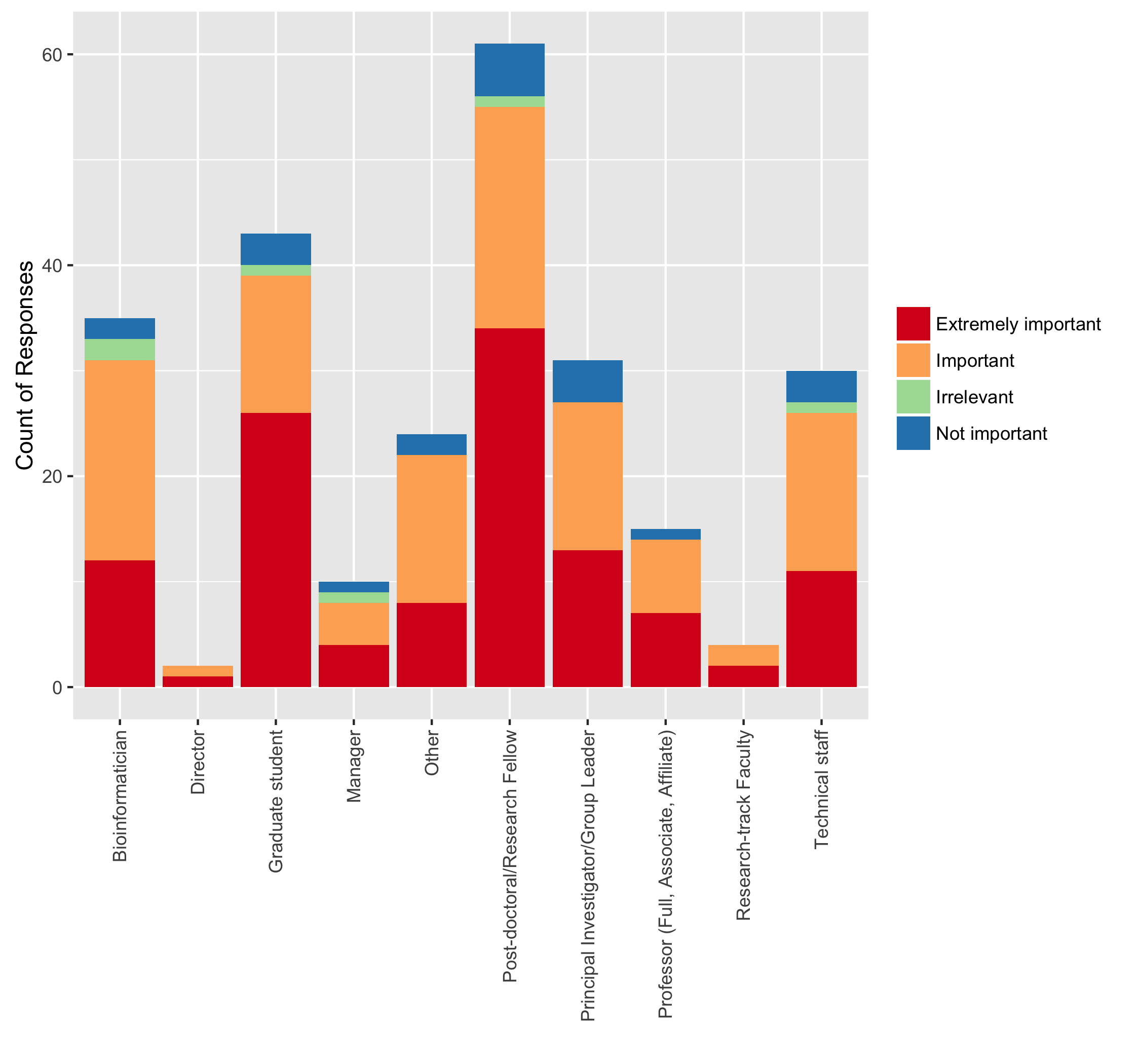

Supplement: S15 Fig — (TIF) [file pcbi.1004916.s016.tif]

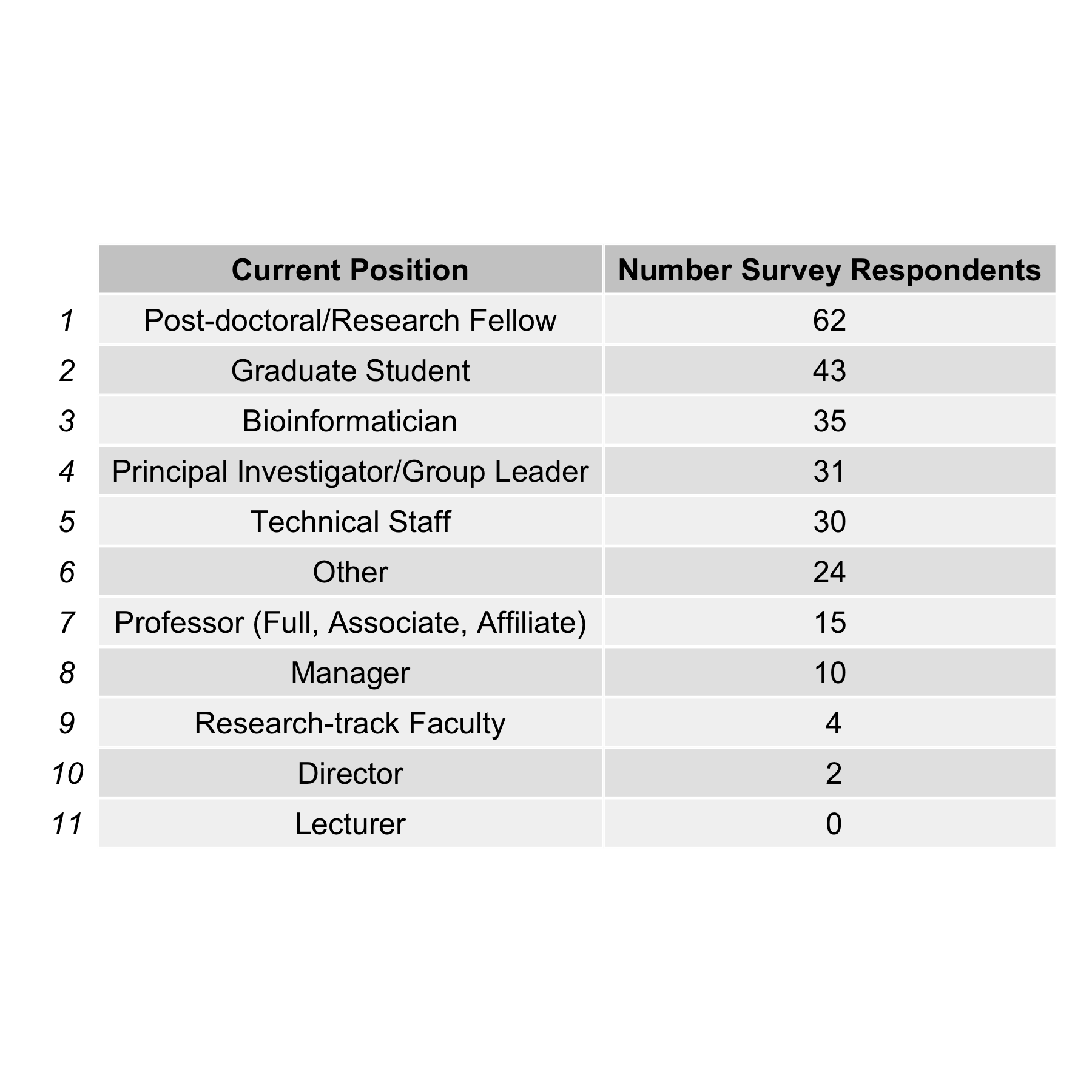

Supplement: S1 Table — (TIF) [file pcbi.1004916.s017.tif]

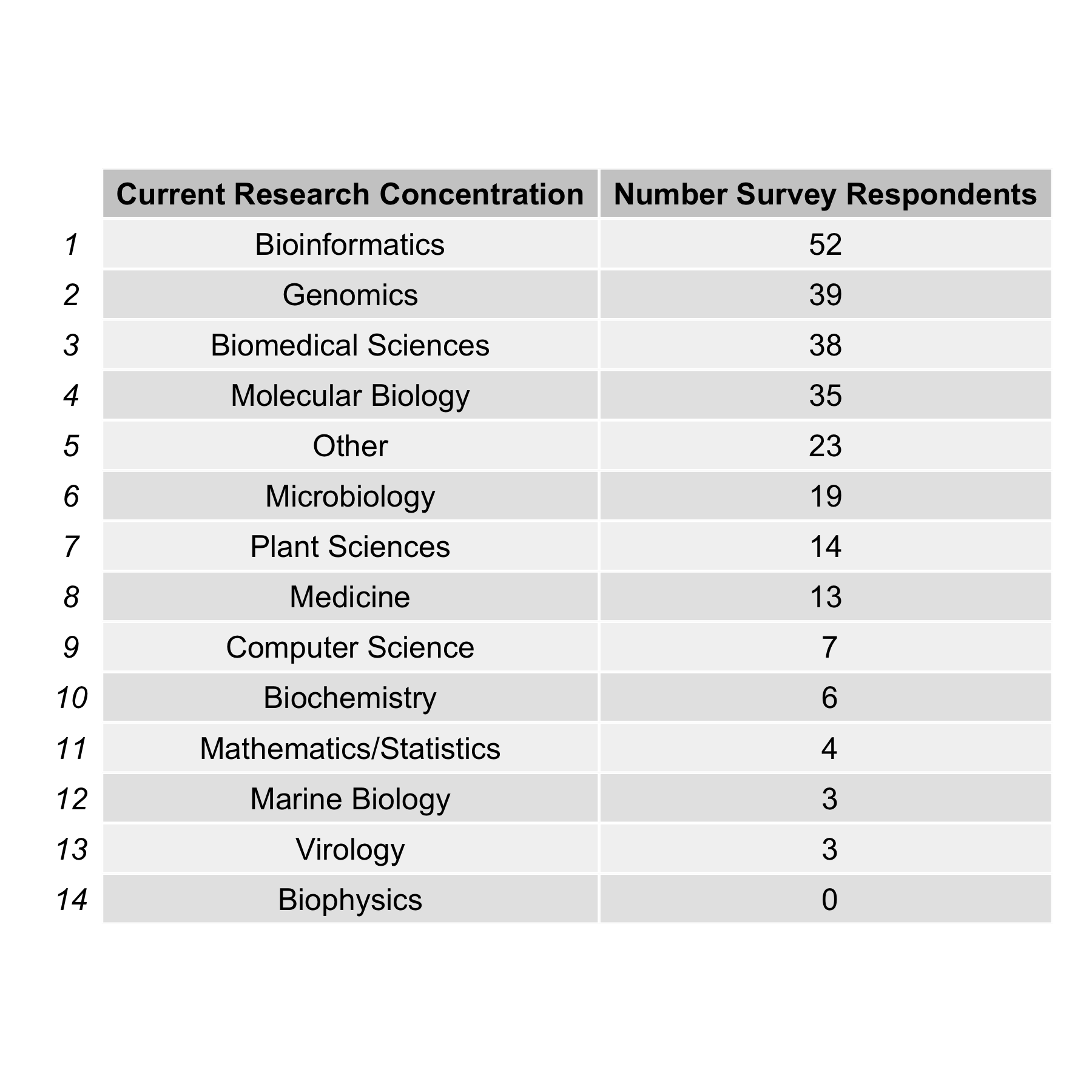

Supplement: S2 Table — (TIF) [file pcbi.1004916.s018.tif]

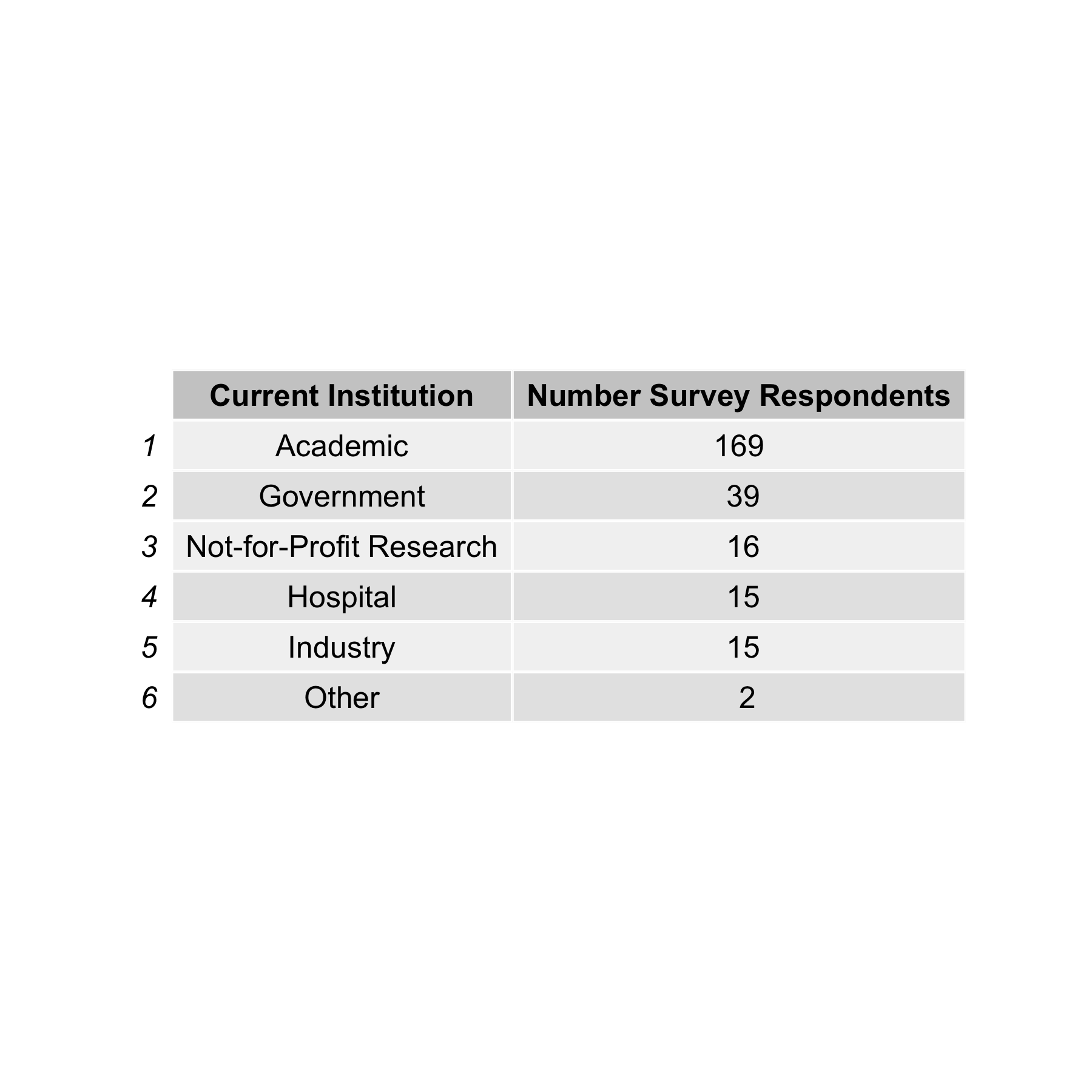

Supplement: S3 Table — (TIF) [file pcbi.1004916.s019.tif]

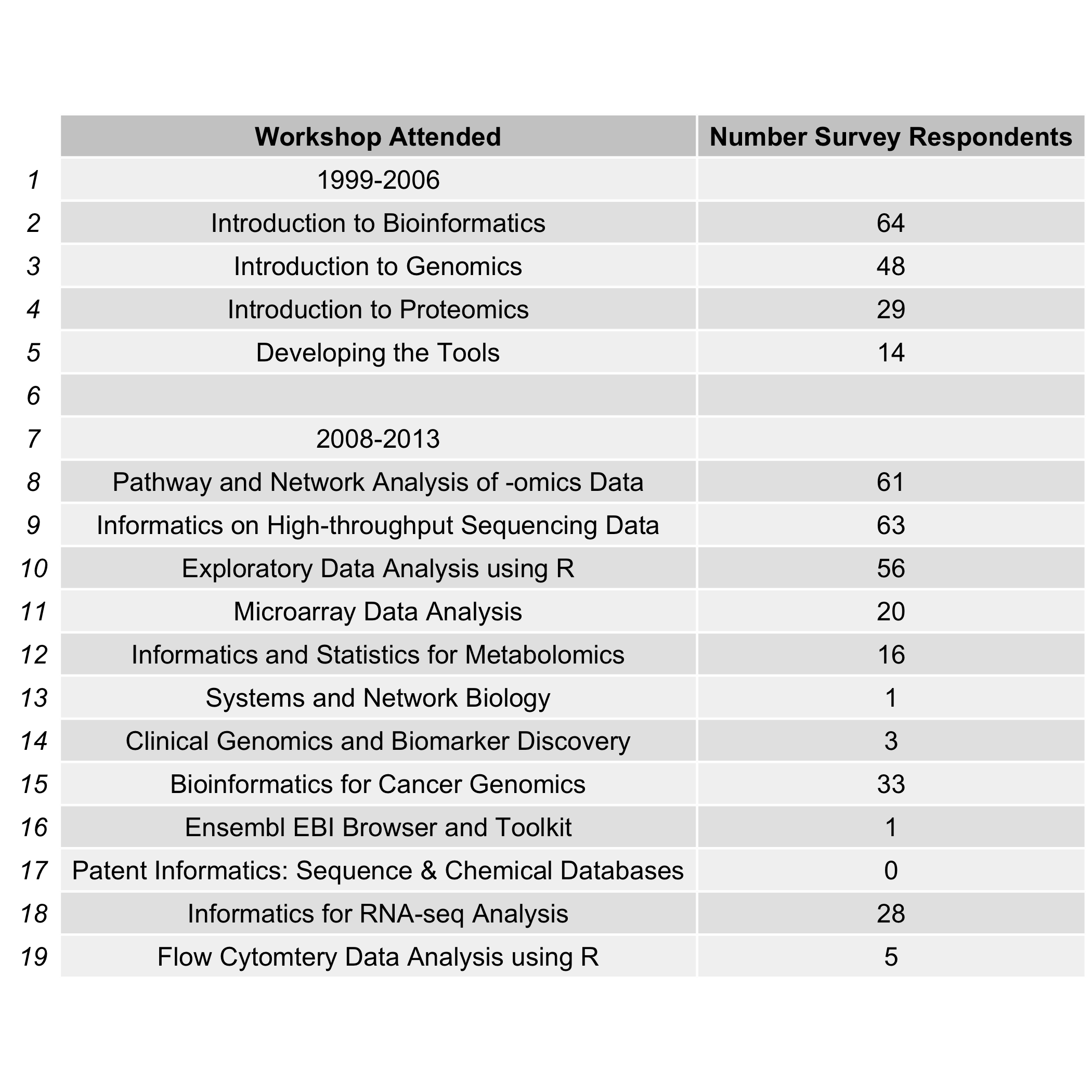

Supplement: S4 Table — (TIF) [file pcbi.1004916.s020.tif]
